# Supplementary material for: Human iPSC-derived preclinical models to identify toxicity of tumor-specific T cells with clinical potential
Source: Mol Ther Methods Clin Dev. 2023 Jan 20;28:249–61. doi: 10.1016/j.omtm.2023.01.005 (PMC9931760; doi:10.1016/j.omtm.2023.01.005)
Supplement: Document S1. Figures S1–S4 and Tables S1 and S2 [file mmc1.pdf]

OMTM, Volume *28*

Supplemental information

Human iPSC-derived preclinical models to identify toxicity of tumor-specific T cells with clinical potential

Rosa A. van Amerongen, Laura T. Morton, Umesh G. Chaudhari, Dennis F.G. Remst, Renate S. Hagedoorn, Cathelijne W. van den Berg, Christian Freund, J.H. Frederik Falkenburg, and Mirjam H.M. Heemskerk

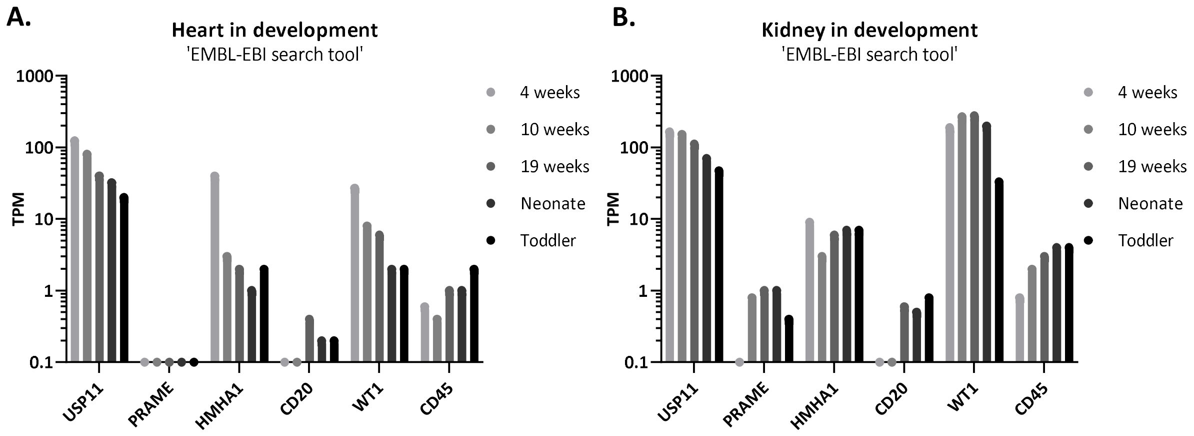

Figure S1: Expression of the T-cell targets in kidney and heart during development

Public available RNAseq gene expression data of the T-cell targets in heart **(A)** and kidney **(B)** tissue during development, given in transcripts per million (TPM). The data is extracted from the online available EMBL-EBI search tool in dataset: 'Human RNA-seq time-series of the development of seven major organs'.[30] Two or three samples are shown per time frame, with three timepoints after fertilization (4, 10 and 19 weeks), one neonatal timepoint (heart samples = 0, 6 or 94 days, kidney samples = 0, 18 or 34 days) and one toddler timepoint (heart and kidney samples = 2 and 4 years). Since HMHA1 and CD20 are hematopoietic-restricted marker, expression of the hematopoietic-restricted marker CD45 is included as a measure for the presence of hematopoietic cells in these samples.

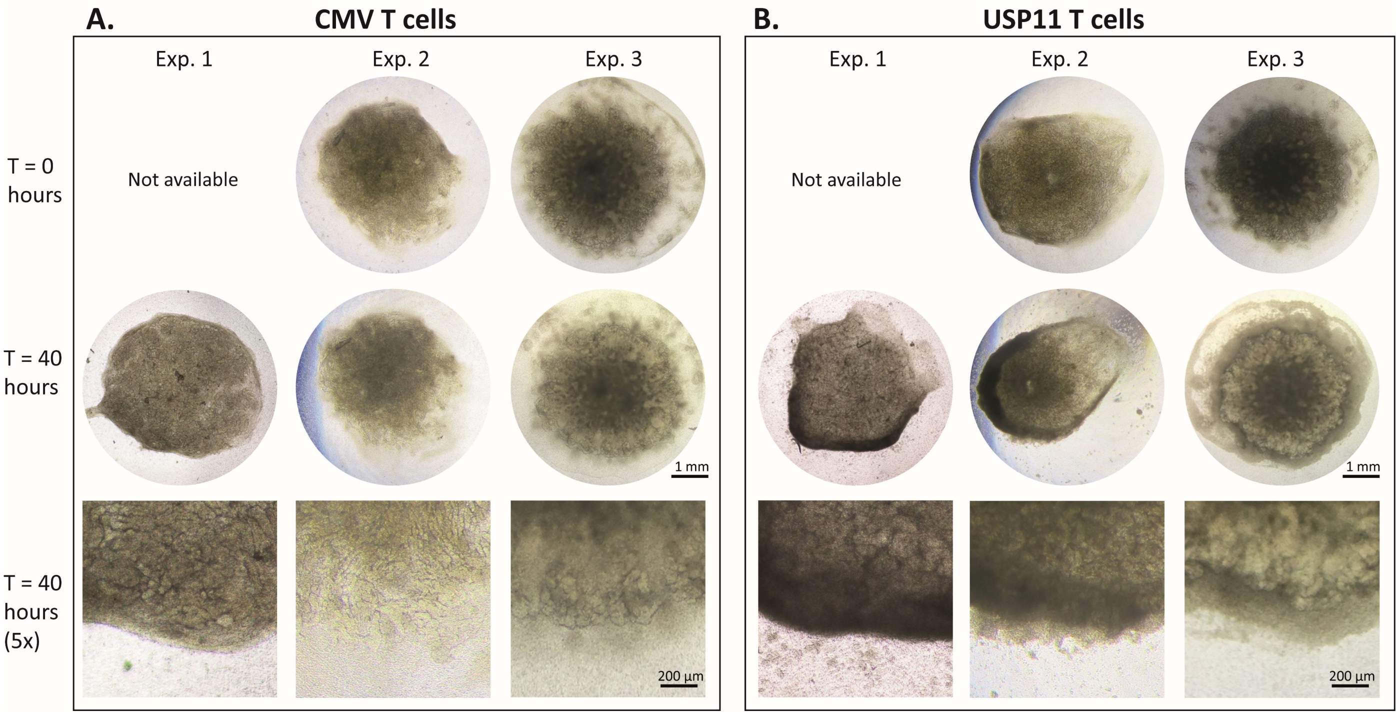

Figure S2: Transmission light images of organoids cocultured with CMV and USP11 T cells

Transmission light images of organoids at timepoint 0 and after 40 hours of coculture with CMV T cells (**A**) or USP11 T cells (**B**). Shown are representative images for three independent experiments (Exp.). Per experiment three organoids were included for each T-cell product. The 5x images shown for T = 40 zoom in on the edges of the organoids.

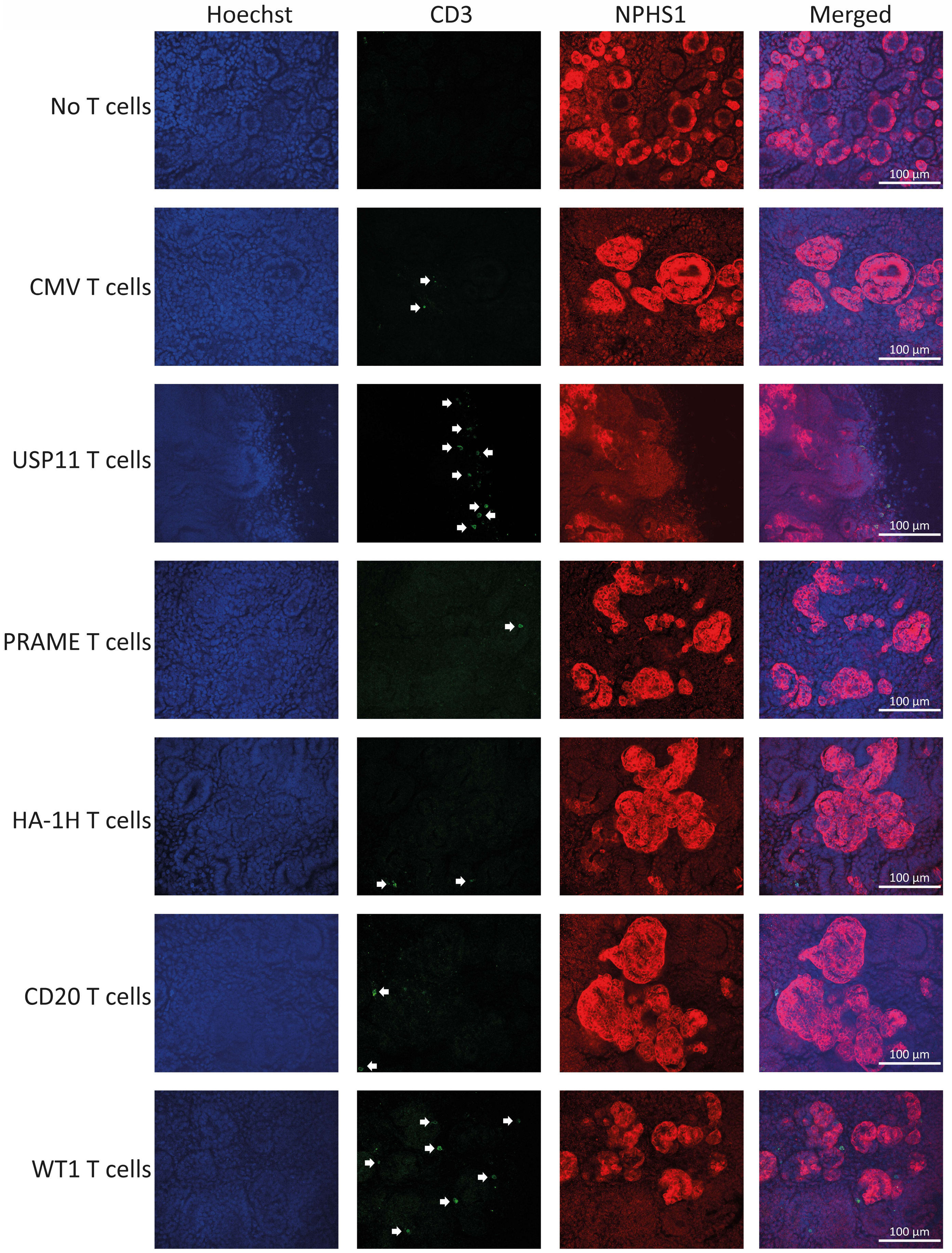

Figure S3: Immunofluorescent images of the kidney organoids cultured with the different T-cell products

Immunofluorescent images of the organoids after 40 hours of coculture with the different T cells, showing glomerular structures (NPHS1), T cells (CD3) and a counterstaining with Hoechst (nuclei). White arrows mark T cells. The images show a variation in number of infiltrated T cells and location of the T cells. 10 images were captured per organoid, representative images are shown.

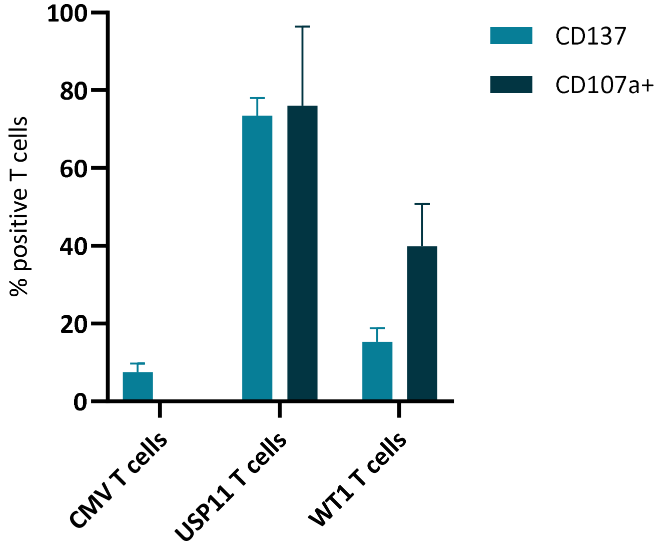

Figure S4: CD137 and CD107a expression on CD8+ T cells infiltrated in hiPSC-derived kidney organoids

The combined percentages of CD137 and CD107a positive infiltrated CD8+ T cells after 40 hours of coculture with the hiPSC-derived kidney organoids. Shown is data of one experiment, two organoids were dissociated for each T-cell product. Values and error bars represent mean and SD of the two organoids.

Table S1. Antibodies used for flow cytometry

Antibody	Conjugate	Company	Article number	Dilution
CD8	Alexa Fluor 700	Invitrogen	MHCD0829	1:300
Murine TCR-Cβ	APC	BD Pharmingen	553174	1:300
Human TCR-αβ	PerCP-Cy5.5	BioLegend	306724	1:150
pMHC-multimers	PE	In house	x	1:300
CD8	PE	BD Pharmingen	555367	1:960
CD137	APC	BD Pharmingen	550890	1:60
CD107a	BV 421	BD Horizon	562623	1:28
CD54	APC	BioLegend	353112	1:240
HLA-A*02:01	PE	BD Pharmingen	558570	1:300
Cardiac Troponin T	VioBlue	Miltenyi Biotec	130-120-402	1:50
T-box18 (IgG)	x	Sigma-Aldrich	HPA029014	1:100
IgG antibody	Alexa Fluor 488	Invitrogen	A11034	1:200

Table S2. Antibodies used for immunofluorescence of hiPSC-derived kidney organoids

Antibody name	Conjugate/Species	Company	Article number	Dilution
NPHS1	Sheep	R&D Systems	AF4269	1:100
CD3	Mouse	Dako	M7254	1:25
Hoechst33258	x	Thermo Fisher Scientific	H3570	1:10,000
Donkey-α-Sheep	Alexa Fluor 647	Thermo Fisher Scientific	A-21448	1:500
Donkey-α-Mouse	Alexa Fluor 488	Thermo Fisher Scientific	A-21202	1:500
