# Supplementary material for: Human iPSC-derived preclinical models to identify toxicity of tumor-specific T cells with clinical potential
Source: Mol Ther Methods Clin Dev. 2023 Jan 20;28:249–61. doi: 10.1016/j.omtm.2023.01.005 (PMC9931760; doi:10.1016/j.omtm.2023.01.005)
Supplement: Document S2. Article plus supplemental information [file mmc2.pdf]

Molecular Therapy
Methods & Clinical Development
Original Article

Human iPSC-derived preclinical models to identify toxicity of tumor-specific T cells with clinical potential

Rosa A. van Amerongen,[1] Laura T. Morton,[1] Umesh G. Chaudhari,[2] Dennis F.G. Remst,[1] Renate S. Hagedoorn,[1] Cathelijne W. van den Berg,[3] Christian Freund,[2] J.H. Frederik Falkenburg,[1] and Mirjam H.M. Heemskerk[1]

[1]Department of Hematology, Leiden University Medical Center, 2333ZA Leiden, the Netherlands; [2]LUMC hiPSC Hotel, Department of Anatomy and Embryology, Leiden University Medical Center, 2333ZA Leiden, the Netherlands; [3]Department of Internal Medicine-Nephrology and Einthoven Laboratory of Vascular and Regenerative Medicine, Leiden University Medical Center, 2333ZA Leiden, the Netherlands

The balance between safety and efficacy of T cell therapies remains challenging and T cell mediated toxicities have occurred. The stringent selection of tumor-specific targets and careful selection of tumor-specific T cells using T cell toxicity screenings are essential. *In vitro* screening options against vital organs or specialized cell subsets would be preferably included in preclinical pipelines, but options remain limited. Here, we set up preclinical models with human induced pluripotent stem cell (hiPSC)-derived cardiomyocytes, epicardial cells, and kidney organoids to investigate toxicity risks of tumor-specific T cells more thoroughly. CD8+T cells reactive against PRAME, HA-1H, CD20, or WT1, currently used or planned to be used in phase I/II clinical studies, were included. Using these hiPSC-derived preclinical models, we demonstrated that WT1-specific T cells caused on-target toxicity that correlated with target gene expression. Multiple measures of T cell reactivity demonstrated this toxicity on the level of T cells and hiPSC-derived target cells. In addition, phenotypic analysis illustrated interaction and crosstalk between infiltrated T cells and kidney organoids. In summary, we demonstrated the benefit of hiPSC-derived models in determining toxicity risks of tumor-specific T cells. Furthermore, our data emphasizes the additional value of other measures of T cell reactivity on top of the commonly used cytokine levels.

INTRODUCTION

The use of T cell based therapies for the treatment of both hematological and solid tumors is rapidly increasing, and has illustrated clinical efficacy.[1–3] However, several of the pioneering T cell based therapies have demonstrated that the balance between therapeutic efficacy and safety remains a challenge as T cell mediated toxicities have occurred.[4] Although different strategies to manage acute toxicities are available, preventing toxicity of T cell based therapies by preclinical analyses is without doubt the safest strategy. The stringent selection of appropriate tumor-specific targets and the careful selection of tumor-specific T cells with a highly specific recognition pattern are essential to prevent on-target off-tumor and off-target toxicities, respectively.

When the tumor target is also expressed in healthy tissues, on-target off-tumor toxicity may occur, and expression in vital organs may lead to life-threatening toxicity. Selection of tumor targets with highly tumor-restricted expression patterns, deduced from the publicly available gene expression databases is essential in preventing toxicity. Although databases with thousands of samples across a wide variety of healthy tissues are available, gene expression data of specialized tissues and cell subsets remain limited.[2,5] For instance, unknown MAGE-A12 expression in a cell subset present in brain tissue resulted in severe neurological on-target off-tumor toxicity following anti-MAGE-A3/A9/A12 T cell receptor (TCR) gene therapy.[6] In addition, even if gene expressions are known, estimating cutoff gene expression values resulting in absence of T cell reactivity is difficult and very complex to predict, since reactivity is dependent on affinity of the TCR and avidity of the interaction between engineered T cells and target tissues.[7] For example, although expression of carcinoembryonic antigen (CEA) in colonic crypts was known but estimated to be too low to induce severe toxicity, a CEA-reactive TCR resulted in unwanted severe toxicity.[8]

These clinical examples illustrate the need for thorough toxicity screenings in the preclinical pipeline of T cell based therapies. On top of stringent selection of tumor targets and their respective tumor-specific T cells, T cell reactivity screenings against vital organs would preferably be included in preclinical pipelines. Unfortunately, *in vitro* screening options using vital organs are limited, and toxicity risks associated with specialized or understudied cell subsets may potentially be missed. Human induced pluripotent stem cells (hiPSCs) can be differentiated into specific vital tissue organoids or unique vital cell subsets may be a valuable addition to overcome these limitations. Initially these hiPSC-derived models showed their value in disease modeling, drug discovery, and toxicity screenings.[9–12] In

Received 2 September 2022; accepted 17 January 2023;
https://doi.org/10.1016/j.omtm.2023.01.005.

Correspondence: Mirjam H.M. Heemskerk, Department of Hematology, Leiden University Medical Center, 2333ZA Leiden, the Netherlands.
E-mail: m.h.m.heemskerk@lumc.nl

This is an open access article under the CC BY license (http://creativecommons.org/licenses/by/4.0/).

Table 1. Characteristics of the included tumor-specific T cell receptors and T cell clones

Name	Target gene	Peptide	Initial T cell clone
CMV TCR	*HCMV* AD169	NLVPMVATV	AV18/BV13 Heemskerk et al.[22]
USP11 TCR	*USP11*	FTWEGLYNV	HSS12 Amir et al.[21]
PRAME TCR	*PRAME*	SLLQHLIGL	HSS1 Amir et al.[17]
HA-1H TCR	*HMHA1*	VLHDDLLEA	HA1.M7 Marijt et al.[24]
CD20 TCR	*CD20*	SLFLGILSV	1E9 Jahn et al.[19]
WT1 T cell clone	*WT1*	ALLPAVPSL	22.1H1 van Amerongen et al.[20]

For each T cell type the targeted gene, targeted peptide, and the name of the initial T cell clone are listed. The references refer to the articles in which the T cell clones were initially identified.

addition, patient-derived tumor organoids have shown their potential in studying tumor-specific immune reactivity and have demonstrated translational applications. These organoids permitted the isolation of tumor-reactive T cells from autologous tumor organoids as well as the assessment of cytotoxic efficacy of engineered T cells.[13–15]

In this study, we established hiPSC-derived preclinical models for different heart cell types as well as kidney organoids to investigate the potential of these models in detecting T cell mediated toxicity. Both models represent specialized cell subsets and tissues without preclinical options to investigate toxicity risks of T cells. Using different tumor-specific T cells with clinical potential, we demonstrated with multiple measures of T cell reactivity that the developed hiPSC-derived preclinical models can be used to determine toxicity risks of T cell based therapies.

RESULTS

Generation and validation of tumor-specific T cells

TCR-transduced CD8 T cell products (TCR-T cells) with five different antigen specificities and a T cell clone were generated to test the applicability of the hiPSC-derived preclinical models (Table 1). Two of the TCRs are currently investigated in phase I/II clinical studies, one targeting the minor histocompatibility antigen (MiHA) HA-1H,[16] encoded by the HMHA1 gene, and the other targeting tumor-associated antigen PRAME.[17,18] TCRs targeting B cell specific antigen CD20[19] or T cells targeting tumor-associated antigen WT1,[20] which are still in preclinical phase, were also used. Based on previous extensive *in vitro* and *in vivo* studies, these T cells were considered highly potent and antigen-specific. The TCR recognizing housekeeping gene USP11 was included as a positive control[21] and the human CMV-reactive TCR was included as negative control.[22] Each T cell population recognizes a peptide presented in HLA-A*02:01. For the generation of the TCR-T cells, CD8+ T cells were isolated from healthy donors and stimulated (Figure 1A). To exclude reactivities of the endogenous TCR or mispairing with the endogenous TCR, both endogenous TCRα and TCRβ genomic sequences were edited 1 day prior to the TCR transduction using CRISPR-Cas9.[23] This resulted in a double knockout (KO) of the human TCR (hTCR) in $\geq$94% of the CD8 cells. Each transgenic TCR (tgTCR) was murinized (mTCR) to allow distinction between the introduced and endogenous TCR and mTCRs were transduced using retroviral vectors into CD8+ T cells. Four days after transduction, the CD8 cells were enriched for mTCR, and flow cytometry analysis at day 10 after isolation demonstrated that $\geq$93% of the CD8 cells were mTCR+ hTCR− (Figure 1B).

All T cell products specifically stained with their corresponding peptide-MHC (pMHC)-multimer (Figure 1C). Functionality of the T cells was assessed by interferon (IFN)-γ production following overnight coculture, and antigen-specific reactivity of the different T cell products correlated with specific target gene expression in the different target cell lines (Figures 1D and 1E). Previously, all T cell products demonstrated antigen-specific cytotoxicity of tumor cells.[17,19,20,24] In summary, these data confirm the antigen specificity and functionality of the six T cell products included in the screenings of the hiPSC-derived preclinical models.

T cell reactivity against hiPSC-derived heart cells

To investigate T cell mediated heart toxicity by the tumor-specific T cells, two crucial and specialized heart cell types, e.g., cardiomyocytes and epicardial cells, were included. hiPSC line LUMC0030iCTRL12 was used for differentiation into cardiomyocytes and epicardial cells, as previously described (Figure 2A).[25,26] This line expresses HLA-A*02:01 and MiHA HA-1H, and was originally derived from skin fibroblasts of a healthy adult donor. For differentiation of the hiPSCs, cells were cultured with a combination of cytokines and small molecules to induce mesoderm formation in 3 days. Addition of WNT inhibitor XAV939 resulted in spontaneously contracting cardiomyocytes from day 6 onward. At day 12 of differentiation, cardiomyocytes were metabolically enriched by culturing for 4 days in media containing lactate and deprived of glucose. Flow cytometric analyses confirmed that 96% of the cells were positive for cardiomyocyte marker Troponin T (Figure 2B). We previously demonstrated by immunofluorescence that these cells also express cardiac sarcomeric markers α-actinin, MLC2a, and MLC2v.[25] For hiPSC-derived epicardial cells, the 3 days of mesoderm induction was also the starting point. The induced cells were cultured for an additional 9 days with different mixtures of cytokines and small molecules, leading to a highly pure population of epicardial cells. Flow cytometric analyses confirmed that 99% of the cells were positive for epicardial marker T-box18+ (Figure 2C). The (pro)epicardial markers COUP-TFII, TCF21, and WT1 were previously demonstrated to be expressed as well, by quantitative polymerase chain reaction (qPCR) and/or immunofluorescence.[26]

By qPCR we quantified relative gene expression of the T cell target genes compared with three housekeeping genes. T cell target gene *WT1* was highly expressed in the epicardial cells (128%), whereas only low expression was observed in cardiomyocytes (0.4%) (Figures 2D and 2E). WT1 is a (pro)epicardial marker, highly expressed during embryonic development, but also detected in adult heart during tissue maintenance and recovery.[27–29] Housekeeping gene *USP11* was expressed in both cardiomyocytes (270%) and

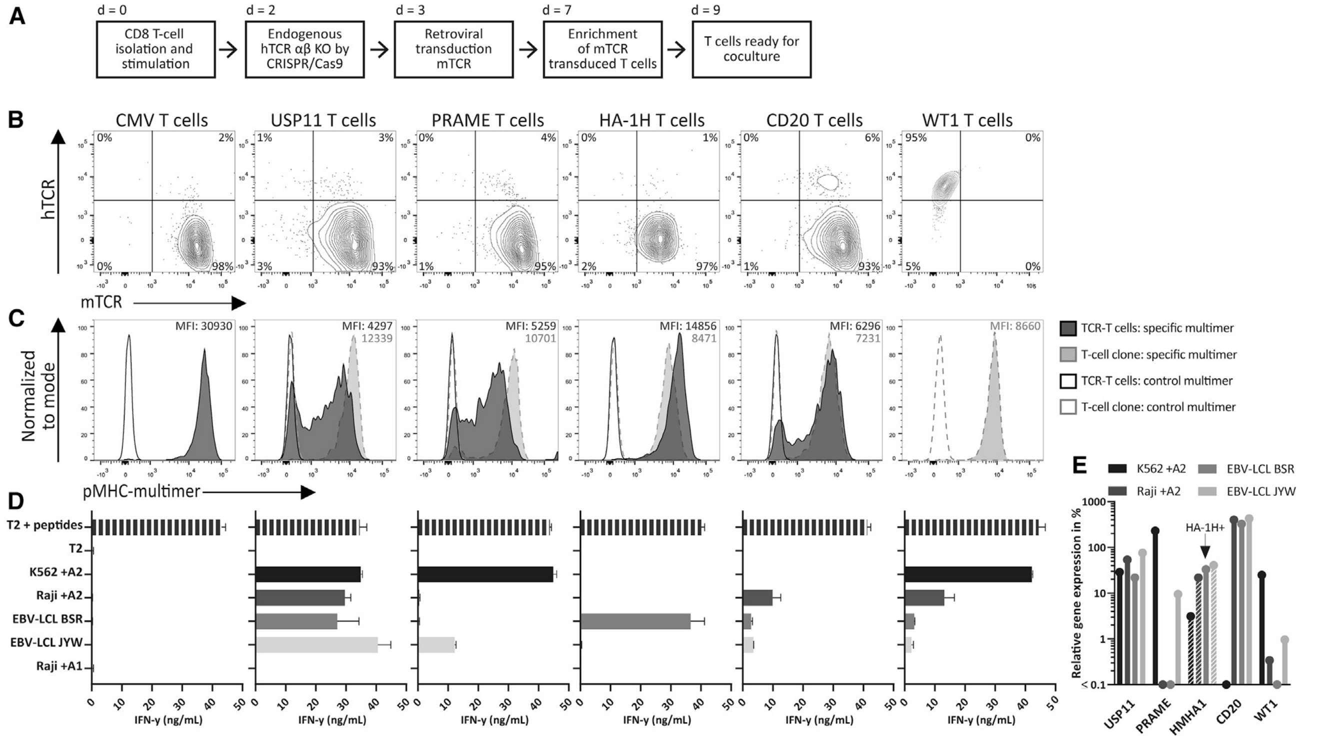

Figure 1. Generation and antigen-specific characteristics of the TCR-T cell products

(A) Schematic of the 10-day production protocol to generate TCR-transduced T cells (TCR-T cells), including knockout (KO) of the endogenous TCRαβ on day 2. The WT1 T cells were restimulated 10 days before use. (B) Representative flow cytometry plots of human (h) TCR and murine (m) TCR expression on T cells, day 10 post isolation. (C) Representative flow cytometry plots of the specific and a control pMHC-multimer on the TCR-T cells and their parental T cell clones, day 10 post isolation. Depicted are mean fluorescence intensity (MFI) values of the TCR-T cells (dark gray) and their parental T cell clones (light gray). (D) IFN-γ production (ng/mL) by the T cells (5,000/well) after overnight coculture with different cell lines (30,000/well). All cell lines were HLA-A*02:01 positive. If cell lines were HLA-A*02:01 negative, the HLA-allele was introduced by transduction (shown as + A2). T2 cells were loaded with a mixture of the targeted peptides (1 μM). Data are representative of two independent experiments; values and error bars represent mean and SD of technical duplicates. (E) The relative gene expression of the target genes in the cell lines, shown as percentage relative to the three housekeeping genes GUSB, VPS29, and PSMB4, which was set at 100%. The minimum gene expression is set at 0.01%. One of the four cell lines expresses the MiHA HA-1H, as indicated by a black arrow.

epicardial cells (169%). Furthermore, only limited *HMHA1* expression was observed in the cardiomyocytes (0.6%) and epicardial cells (0.6%). The observed expression of *WT1* and *HMHA1* correlated with expression of these genes in early embryonic heart (Figure S1A),[30] and we therefore anticipate that hiPSC-derived models may have immature characteristics.

Once differentiated, the hiPSC-derived cardiomyocytes and epicardial cells were cocultured, in two seeding densities, with the different T cell products. After 16 h of coculture, microscopic analysis revealed that the cardiomyocytes remained unharmed and were still regularly contracting when cocultured with CMV, PRAME, HA-1H, CD20, and WT1 T cells. In contrast, coculture with the positive control, the USP11 T cells, led to significant cardiomyocyte detachment indicating cell death (Figure 3A). All non-adherent cells (T cells and cell debris) were removed and DAPI staining was used to quantify the remaining attached cells to quantify the percentage of killed cells (Figures 3B and 3D). On average, 87% ± 7% of the cardiomyocytes were killed after coculture with USP11 T cells (Figure 3D). Coculture of the epicardial cells with the different T cell products demonstrated high killing potential of the USP11 T cells (96% ± 2%) as well as WT1 T cells (94% ± 4%), which correlated with target gene expression (Figure 2E). In line with these findings, activation marker CD137 and degranulation marker CD107a were strongly upregulated on the USP11 T cells cocultured with both hiPSC-derived heart cell types, while WT1 T cells only upregulated these markers when cocultured with *WT1* expressing epicardial cells (Figures 3C and 3E). The activated USP11 and WT1 T cells also produced significant amounts of the pro-inflammatory cytokine IFN-γ, as measured by ELISA (Figure 3F).

Some killing of epicardial cells was observed (9% ± 11%, Figure 3D) by the HA-1H T cells at the highest seeding density, which correlated with CD137 upregulation on the HA-1H T cells (15% ± 3%, Figure 3E). Compared to the positive control USP11 T cells this upregulation was limited and no IFN-γ production or upregulation of degranulation marker CD107a was observed (Figures 3E and 3F). Based on the limited reactivity and the quantified 0.6% *HMHA1*

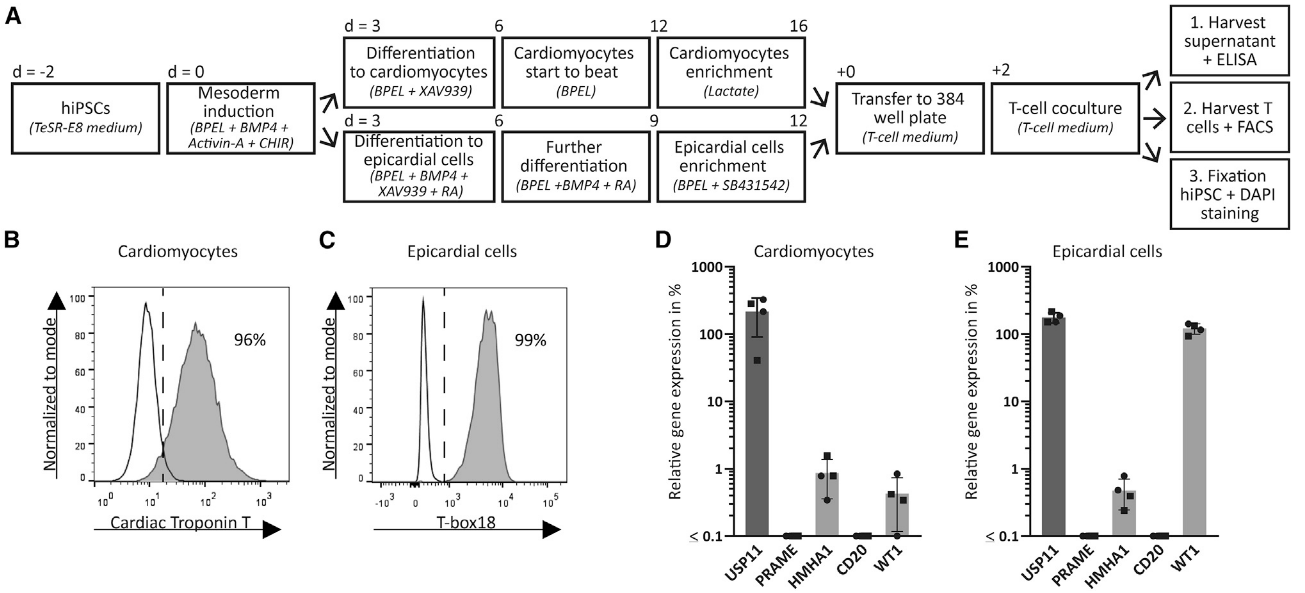

Figure 2. Characteristics of the hiPSC-derived heart cells

(A) Schematic of the protocol to generate cardiomyocytes and epicardial cells, combined with the coculture assay with the T cells. Medium and supplements per step are shown between brackets. (B) Flow cytometry plots showing the differentiation purity of the cardiomyocytes (Cardiac Troponin T). (C) Flow cytometry plots showing the differentiation purity of the epicardial cells (T-box18). (D) Relative gene expression of the target genes in cardiomyocytes. (E) Relative gene expression of the target genes in epicardial cells. (D and E) Gene expression is shown as percentage relative to housekeeping genes GUSB, VPS29, and PSMB4, which was set at 100%. The minimum gene expression is set at 0.01%. Symbols represent averaged triplicate values of cells derived from two different hiPSC lines in two independent experiments; values and error bars depict mean and SD. Round symbols represent the hiPSC line included in the T cell coculture experiments.

expression in these hiPSC-derived epicardial cells (Figure 2E), we assume this low reactivity was the result of on-target off-tumor reactivity.

To summarize, T cell reactivity observed, as demonstrated by cytotoxicity of heart cells, upregulation of activation markers and cytokine production by T cells, correlated with target gene expression (*USP11*, *WT1*, and *HMHA1*), indicating that on-target off-tumor T cell reactivity was clearly detectable in this model. Overall, our data demonstrate the hiPSC-derived cardiomyocytes and epicardial cells can be used to investigate toxicity risks of tumor-specific T cells.

T cell reactivity against hiPSC-derived kidney organoids

To investigate T cell mediated kidney toxicity, we used instead of single cell populations the complete hiPSC-derived kidney organoids (Figure 4A).[31] hiPSC line LUMC0072iCTRL01 was used for this study, which expressed HLA-A*02:01 and was originally derived from skin fibroblasts from a healthy adult donor. hiPSCs were maintained in Essential-8 and differentiated to primitive streak, followed by induction of intermediate mesoderm. At day 7 of differentiation, cells were dissociated and transferred as pellet onto a transwell membrane to form 3D structures. Self-organized organoids were observed within 25 days of culture, showing segmenting nephron structures with Nephrin (NPHS1) expressing cells, representing the glomeruli (Figures 4B and 4C). Using the same protocol and hiPSC line, we previously demonstrated the presence of podocytes (WT1+), proximal tubules (CUBN+, LTL+), distal tubular structures (ECAD+), and endothelial cells (CD31+) by immunofluorescence.[31] At day 7 + 18, the kidney organoids were cocultured with the different T cell products. After 40 h, whole kidney organoids were stained for NPHS1 and CD3 to visualize the glomerular structures and infiltration of T cells in the kidney organoids (Figure 4C). Kidney organoids that were not cocultured with T cells were dissociated and gene expression of all T cell target genes was quantified by qPCR. Besides housekeeping gene *USP11* (230%), we quantified high *WT1* (205%), low *HMHA1* (2.4%), and low *PRAME* (0.4%) expression (Figure 4D). The expression of *WT1* correlated with previous findings that *WT1* is highly expressed in adult renal podocytes present in the glomeruli.[27,28] The expression of *HMHA1* correlated with low expression in publicly available embryonic kidney (Figure S1B).[30] Furthermore, low expression of *PRAME* correlated with the previously detected low *PRAME* expression in renal proximal tubular epithelial cells.[17]

We did not observe major morphological changes of the kidney organoids cocultured with the CMV, PRAME, HA-1H, CD20, and WT1 T cells (Figure 5A, representative images of six organoids from two independent experiments). Only the kidney organoids cocultured with the USP11 T cells, demonstrated direct damage on the edge of the organoids, depicted by darkened and detached edges (Figure 5A). These darkened and detached edges were detectable in all independent experiments (Figure S2). Immunofluorescent images did not reveal differences in the glomerular structures, but demonstrated a

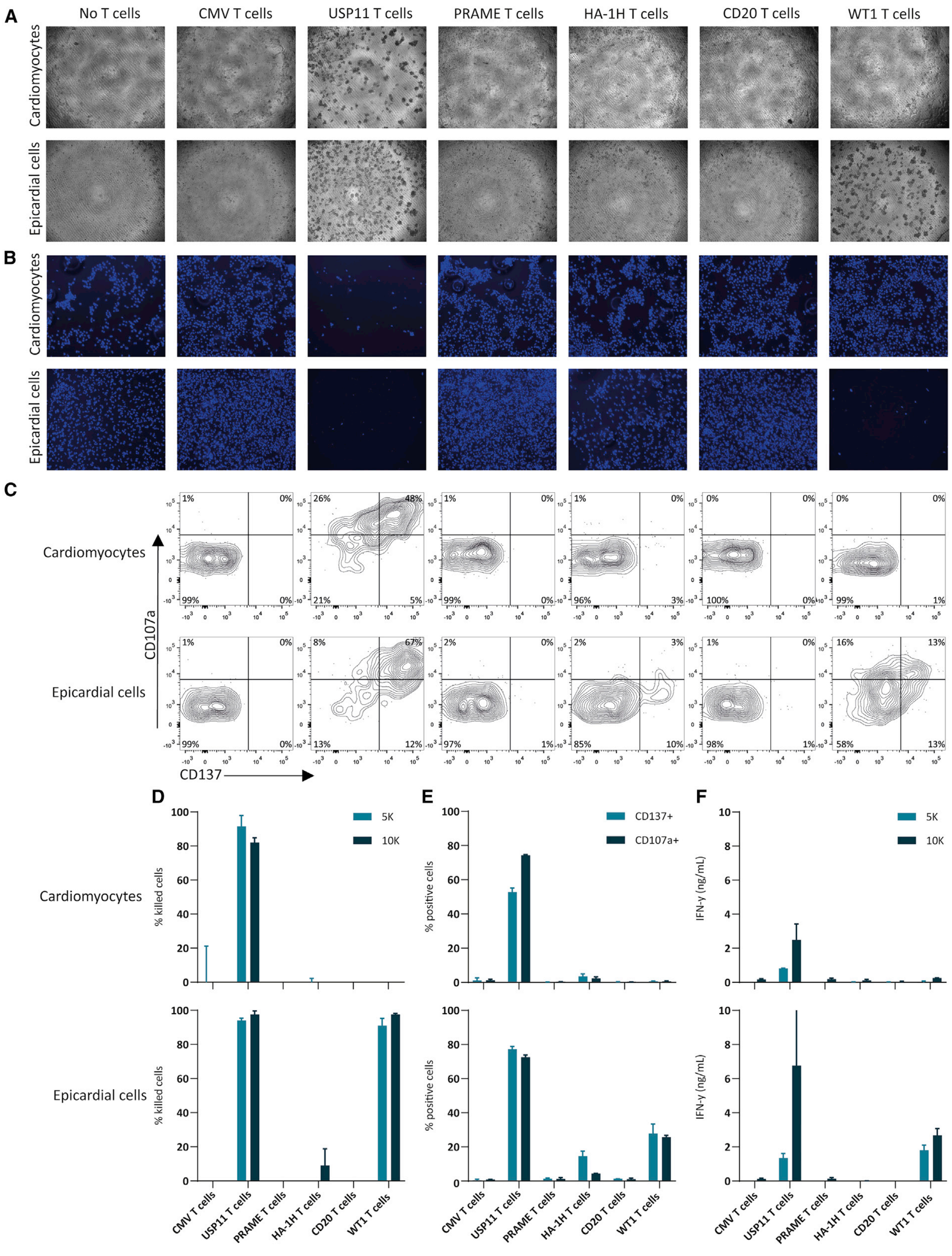

(legend on next page)

trend of increased infiltration of CD3-expressing USP11 and WT1 T cells (Figure S3). Most USP11 T cells were observed on the edges of the organoids, likely due to the homogeneous expression of housekeeping gene *USP11*, whereas the WT1 T cells were mainly found within the organoids, correlating with the previously observed *WT1* expression within the glomeruli.[31]

To further assess T cell reactivity, we dissociated the organoids, resulting in a mixed population of organoid cells (99%) and infiltrated or attached CD8+ T cells (1%) (Figure 5B). No upregulation of the inducible costimulatory receptor CD137 (4-1BB) was observed on the CMV, PRAME, HA-1H, and CD20 T cells. In contrast, over 80% of the USP11 T cells and 50% of the WT1 T cells showed clear CD137 upregulation (Figures 5B and 5D). In addition, 75% of the USP11 T cells and 40% of the WT1 T cells showed clear upregulation of degranulation marker CD107a, whereas no upregulation was observed on CMV T cells (Figure S4). T cell reactivity also induced changes on the organoid cells: in absence of T cells, low expression of HLA-A*02:01 and adhesion molecule CD54 (ICAM-1) was detectable by flow cytometry. Interestingly, after 40 h of coculture with USP11 and WT1 T cells, approximately 60%–70% of the organoid cells upregulated HLA-A*02:01 and CD54 expression (Figures 5C and 5E). No upregulation was observed on the kidney organoids cocultured with other T cells. In line with this finding, only the USP11 and WT1 T cells produced cytokine IFN-γ after 16 and 40 h of coculture (Figure 5F). No IFN-γ was measured in the supernatants of the kidney organoids cocultured with the PRAME, HA-1H, CD20, and CMV T cells.

To summarize, in case of homogeneous expression of the target gene (*USP11*) or localized expression of the target gene within the organoids (*WT1*), T cell reactivity by infiltrated T cells was clearly detectable in the kidney organoids, both on the T cell and organoid level. Although direct damage was only visible in the kidney organoids cocultured with the USP11 T cells, we demonstrate that the WT1 T cells were able to infiltrate into the kidney organoid, upregulate activation markers, produce cytokines, and induce changes in the phenotype of kidney organoid cells. Together our data demonstrate hiPSC-derived kidney organoids can be used to investigate toxicity risks of tumor-specific T cells.

DISCUSSION

We established hiPSC-derived preclinical models to investigate toxicity risks of tumor-specific T cells with clinical potential. These models can fulfill the need for *in vitro* options to examine toxicity risks of T cells directed toward vital organs or specialized cell subsets. By measuring cytokine production, killing potential, and phenotypic analysis of cocultured T cells and hiPSC-derived target organoids/cells, on-target off-tumor toxicity could be determined. In case of expression of the target gene, as demonstrated for housekeeping gene *USP11* and tumor gene *WT1*, T cell reactivity was clearly observed in these models. The reactivity of WT1 T cells toward hiPSC-derived kidney organoids and epicardial cells reflect the immature phenotype of the models, but also indicate possible toxicity risks of WT1-targeting therapies since WT1 expression partly remains or becomes re-activated in the adult situation.

Our hiPSC-derived models demonstrate the added value of multiple measures of T cell reactivity on top of the commonly used cytokine production assays. First, costimulatory receptor CD137, and degranulation marker CD107a gave additional insight in the T cell effector function. Both markers are considered to be more sensitive than cytokine levels alone as not all CD107a and CD137 expressing cells produce IFN-γ, whereas all CD107a-expressing T cells exert cytotoxic activity and all CD137-expressing T cells were antigen specifically activated.[32,33] Second, infiltration of T cells into the kidney organoid and upregulation of HLA class I and CD54 expression on the kidney organoid cells demonstrate interaction and crosstalk between T cells and target cells. Upon recognition of cells within the kidney organoids, activated CD8+ T cells release pro-inflammatory cytokines, such as IFN-γ, that will induce expression of molecules involved in antigen presentation, like HLA and adhesion molecule CD54.[34–36] This cascade of events makes neighboring kidney organoid cells easier to recognize and hypothetically more susceptible to T cell-induced apoptosis.[37] Third, after coculture with USP11 T cells, microscopic analysis of the kidney organoids and DAPI staining of the heart cells showed a direct toxicity effect on the target cells. However, no direct toxicity effect was observed when the kidney organoids were cocultured with the WT1 T cells. Despite this, by looking at the immune reactivity observed in both infiltrated T cells (Figures 5B, 5D, and 5F) and organoid cells (Figures 5C and 5E), we hypothesize that also WT1 T cells induced on-target off-tumor toxicity against cells within the kidney organoids.

Previously, it was feared that kidney-related toxicity may be induced by the PRAME TCR given the low expression of *PRAME* in renal PTECs and reactivity against renal-derived PTEC cell lines *in vitro*.[17] So far, no model was available to further investigate on-target off-tumor toxicity in the kidney. Although proximal tubular cells are represented in the nephron structures of the kidney

Figure 3. T cell reactivity against hiPSC-derived cardiomyocytes and epicardial cells

Shown are data of one experiment, data are representative of two independent experiments (except for B and D). Per experiment technical duplicates of two seeding densities (5K and 10K) were included. (A–C) Representative images/plots for the 10K seeding density, (D–F) values and error bars represent mean and SD of the technical duplicates. (A) Transmission light images of the cardiomyocytes and epicardial cells after 16 h of coculture with the different T cells. (B) DAPI staining of the remaining cells after removal of the non-adherent cells. Six images were taken per well, covering in total 50% of the wells. (C) Flow cytometry plots of CD137 and CD107a expression on the T cells after 16 h of coculture with the heart cells. (D) Percentages of killed hiPSC-derived cells after 16 h of coculture with the 5K and 10K seeding densities. (E) The combined percentages of CD137 and CD107a positive CD8 cells after 16 h of coculture with the 10K seeding density. (F) IFN-γ production after 16 h of coculture with the 5K and 10K seeding densities.

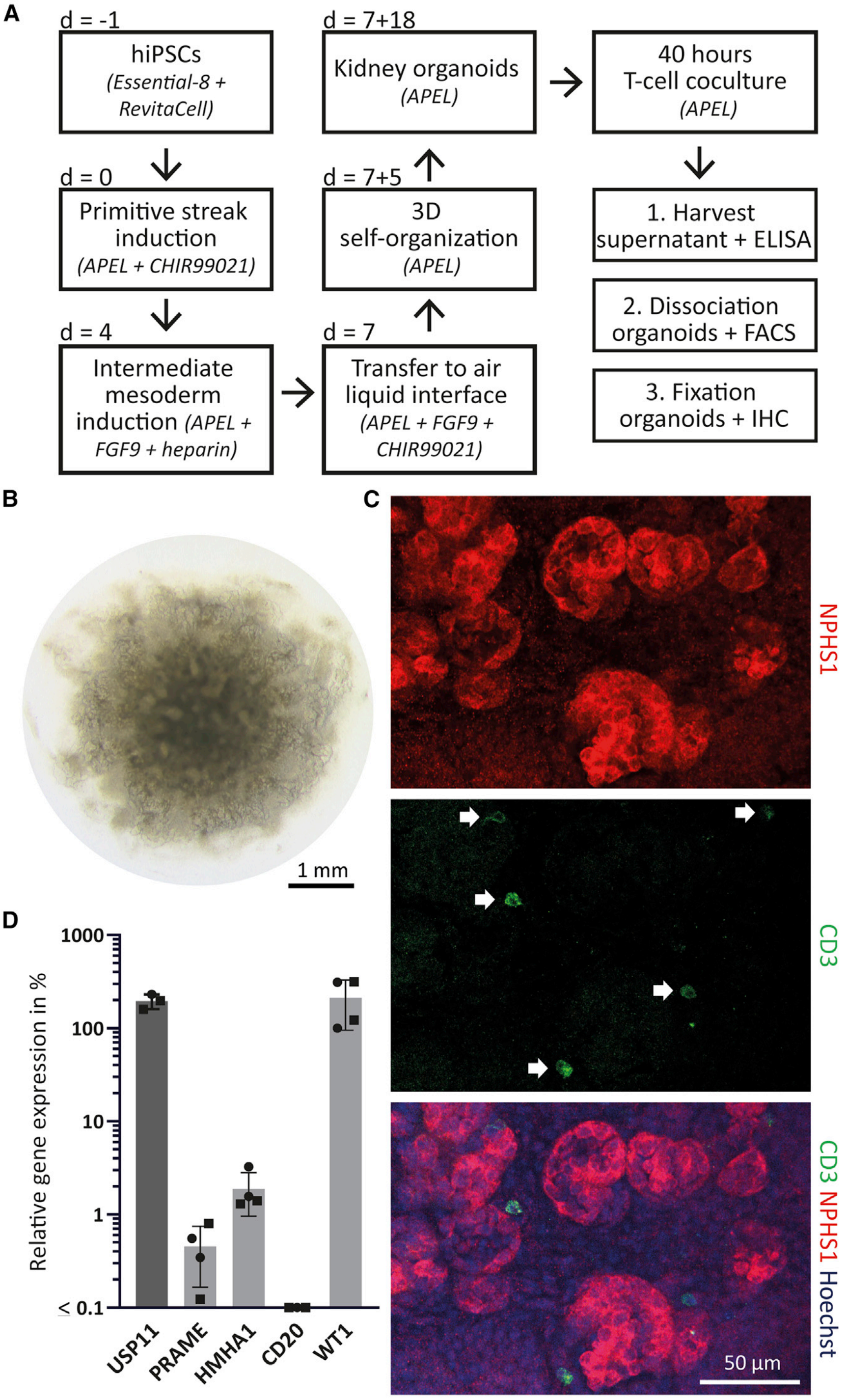

Figure 4. Characteristics of the hiPSC-derived kidney organoids

(A) Schematic of the protocol to generate kidney organoids, combined with the coculture assay with the T cells. Medium and supplements per step are shown between brackets. (B) Representative transmission light image of an organoid at day 7 + 18 of the protocol, with a diameter of ~4–5 mm. (C) Immunofluorescent images of an organoid after 40 h of coculture with the WT1 T cells, showing glomerular structures (NPHS1), infiltrated T cells (CD3) and a counterstaining with Hoechst (nuclei). White arrows mark T cells. (D) Relative gene expression of the target genes in the kidney organoids, shown as percentage relative to housekeeping genes GUSB, VPS29, and PSMB4, which was set at 100%. The minimum gene expression is set at 0.01%. Symbols represent averaged triplicate values of the kidney organoids derived from two different hiPSC lines in two independent experiments; values and error bars depict mean and SD. Round symbols represent the hiPSC line included in the T cell coculture experiments.

organoids, no T cell reactivity was observed for the PRAME TCR. In this study, we have only demonstrated on-target off-tumor toxicity; however, these hiPSC-derived models most likely are also suitable to identify off-target toxicity of T cells. Off-target toxicity can be caused by cross-reactivity of the TCR for a distinct peptide. Previously, this was demonstrated for an affinity-enhanced MAGE-A3 TCR that caused fatal off-target cardiac toxicity. After discontinuing the clinical study, the TCR was screened against hiPSC-derived cardiomyocytes, which were efficiently recognized by the MAGE-A3-specific T cells, and most likely explained the cardiac toxicity.[38] The tumor-specific T cells included in our study were already extensively screened for potential off-target toxicities against a large panel of different healthy cell subsets, but were not tested before against cardiomyocytes, epicardial cells, and kidney organoids.[17,19,20,24]

In vivo mouse models are often suggested to be able to investigate toxicity risks of T cell based therapies due to absence of appropriate alternatives. However, although important information can be obtained from these models concerning effectivity, toxicity measurements are limited since the human proteome as well as the HLA class I and II molecules are not expressed.[5] Making use of hiPSC-derived models to mimic the human situation more accurately might be a better alternative. As previously demonstrated, the hiPSC-derived models closely resemble the different heart cell types and kidney tissue. For the hiPSC-derived cardiomyocytes, typical features were confirmed, e.g., the expression of the cardiac sarcomeric markers Troponin T, α-actinin, MLC2a, and MLC2v, as well as functional properties such as regular spontaneous beating.[25,39] The hiPSC-derived epicardial cells showed similar morphology and marker expression, e.g., T-box18, WT1, COUP-TFII, and TCF21.[26] The self-organized hiPSC-derived kidney organoids were previously

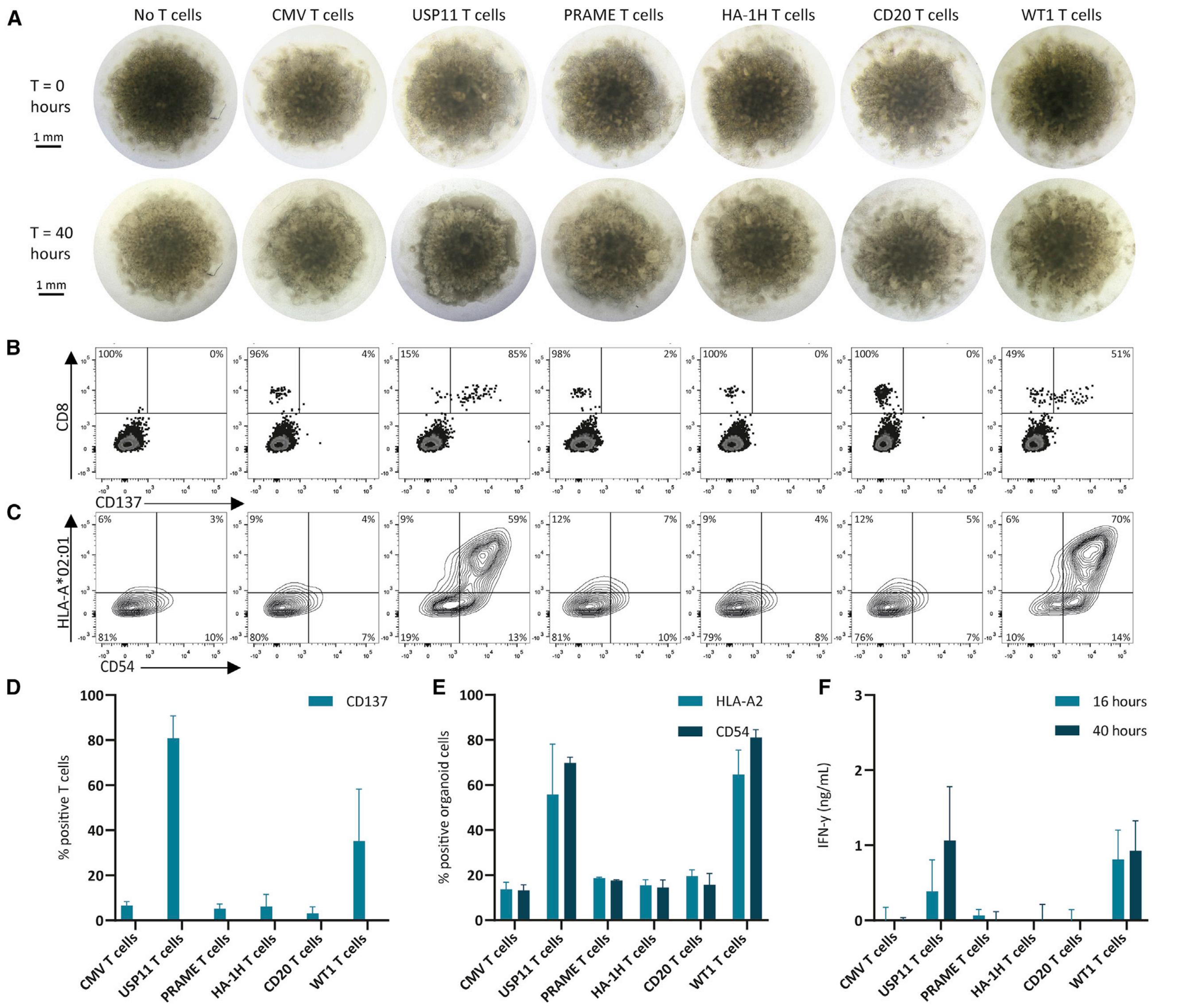

Figure 5. T cell reactivity against hiPSC-derived kidney organoids

Shown are combined data of two independent experiments. Per experiment three organoids were included and two organoids were dissociated separately (for each T cell product). (A–C) Representative images/plots within one experiment, (D–F) data of the technical duplicates of both experiments; values and error bars represent mean and SD. (A) Transmission light images of the organoids at time point 0 and after 40 h of coculture with the different T cell products. (B) Flow cytometry plots of CD8 and CD137 expression on dissociated organoids after 40 h of coculture. The percentages of the CD137 negative and positive cells are shown for the CD8-positive population. The CD8 negative cells are viable organoid cells. (C) Flow cytometry plots of HLA-A*02:01 and CD54 expression on dissociated organoids after 40 h of coculture. (D) The percentages of CD137-positive CD8 cells in the dissociated organoids after 40 h of coculture. (E) The combined percentages of HLA-A*02:01 and CD54 positive organoid cells after 40 h of coculture. (F) IFN-γ production after 16 and 40 h of coculture.

established as models to study nephrotoxicity, since all segmenting nephron structures are present, e.g., glomerular structures (NPHS1+), proximal tubules (CUBN+, LTL+), and distal tubular structures (ECAD+).[9,31] Another advantage of the hiPSC-derived models is the reproducibility, we previously demonstrated with multiple hPSC lines the robustness of the used differentiation protocols as similar marker expression was observed.[25,26,31] The models can also be produced on a large scale and do not have to be generated for each patient separately. An hiPSC bank consisting of well-characterized hiPSC lines expressing all common HLA restriction alleles would be helpful for screening of TCRs with different HLA restrictions. In the future, it would be eminent to include hiPSC-derived 3D heart organoids as an additional preclinical toxicity screening.[40]

To properly interpret the toxicity data, the immaturity of the preclinical hiPSC-derived models has to be taken into account. The

immaturity might result in an underestimation of toxicity risks for genes exclusively expressed in mature cells and an overestimation of toxicity risks for genes involved in embryonic development. The observed high *WT1* and moderate *HMHA1* expression in our hiPSC-derived models correlates with gene expression in embryonic tissues (Figure S1) and highlights the immaturity of the models. WT1 is involved during the embryonic development of the kidneys, gonads, and several organs lined by the mesothelium, such as the heart.[27–29] However, in adults, WT1 is still involved in homeostasis processes for tissue maintenance and recovery, resulting in continuous *WT1* expression in renal podocytes and temporary *WT1* expression in epicardial cells after a myocardial infarct.[27–29] WT1 is considered a safe target for WT1-targeting therapies and no toxicities have been reported[41–43]; however, based on our results, this must be reviewed carefully again. *HMHA1* is a hematopoietic-restricted gene and only in early developing heart *HMHA1* expression is observed that cannot be related to the presence of hematopoietic (CD45+) cells (Figure S1). In adult heart, no *HMHA1* expression above *CD45* expression is observed, and consequently no on-target toxicity by the HA-1H T cells is expected.

In conclusion, the hiPSC-derived preclinical models of specialized cell subsets and tissues described in this study can broaden the preclinical screening pipeline of T cell based therapies and can predict and thereby prevent on- and off-target toxicities of tumor-specific T cells. Our results demonstrate the added value of thorough phenotypic analyses, to determine T cell mediated toxicity on the level of T cells, target cells, and crosstalk between infiltrated T cells and target cells.

MATERIALS AND METHODS

Cell culture and generation and maintenance of hiPSCs

T cells were cultured in T cell medium (TCM) composed of Iscove's Modified Dulbecco's Medium (IMDM) (Lonza), 5% heat-inactivated Fetal Bovine Serum (FBS) (Gibco, Thermo Fisher Scientific), 5% human serum (Sanquin Reagents), 1.5% L-glutamine (Lonza), 1% Pen/Strep (Lonza), and 100 IU/mL IL-2 (Novartis Pharma). 0.2×10^6 T cells were (re)stimulated with 1×10^6 irradiated (35 Gy) PBMCs, 0.1×10^6 irradiated (55 Gy) EBV-LCLs, and 0.8 μg/mL phytohemagglutinin (PHA) (Oxoid Microbiology Products, Thermo Fisher Scientific). The tumor cell lines were cultured in IMDM, 10% FBS, 1.5% L-glutamine, and 1% Pen/Strep and they were tested mycoplasma negative, using the PlasmoTest Mycoplasma Detection Kit (InvivoGen).

hiPSC line LUMC0030iCTRL12 (hPSCregistry line LUMCi004-B), used for the hiPSC-derived cardiomyocytes and epicardial cells, contains the HA-1H miHA and expresses the following HLA alleles: A*02:01, A*24:02/24:353, B*15:01:01, B*40:02/40:356, C*02:02:02, C*03:03/03:357. This hiPSC line was derived from skin fibroblasts of an adult female donor without a known genetic disease. The fibroblasts were reprogrammed by the LUMC hiPSC Hotel using a polycistronic lentiviral vector encoding for Oct4, Sox2, Klf4, and c-Myc as previously described.[44] The hiPSCs were maintained on Vitronectin XF-coated six-well plates (Stem Cell Technologies) in TeSR-E8 medium (Stem Cell Technologies), with daily media changes. Cells were dissociated using Gentle Cell Dissociation Reagent (Stem Cell Technologies) and passaged as small aggregates. hiPSC line LUMC0020iCTRL06 (hPSCregistry line LUMCi028-A),[25] used for additional hiPSC-derived cardiomyocytes and epicardial cells in Figures 2D and 2E, was kindly provided by M. Bellin (Department of Anatomy and Embryology, LUMC, The Netherlands).

hiPSC line LUMC0072iCTRL01 (hPSCregistry line LUMCi029-A), used for the hiPSC-derived kidney organoids, expresses the MiHA HA-1R and the following HLA alleles: A*02:01, A*32:01:01, B*15:18:01, B*15:220, C*07:04/07:181, C*12:03/12:28. This hiPSC line was derived from skin fibroblasts of an adult male donor without a known genetic disease. These fibroblasts were reprogrammed into hiPSCs using the Simplicon RNA Reprogramming Kit (Millipore) (LUMC hiPSC Hotel) as previously described.[31] The hiPSCs were maintained on vitronectin-coated culture dishes in Essential-8 medium (Life Technologies) as small clumps using 0.5 mM UltraPure EDTA (Thermo Fisher Scientific and the day before differentiation as single cells using TrypLE Select (Thermo Fisher Scientific) and the addition of RevitaCell Supplement for 24 h (Thermo Fisher Scientific).[31] All cells included in this research were cultured at 37°C and 5% CO_2.

Gene expression by quantitative polymerase chain reaction

Expression of target genes *PRAME*, *HMHA1*, *CD20*, *WT1*, and *USP11* was quantified by qPCR. Total RNA was isolated using the RNAqueous-Micro Kit (Ambion) or ReliaPrep RNA Cell Miniprep System (Promega). First strand cDNA synthesis was performed with Moloney murine leukemia virus reverse transcriptase and Oligo (dT) primers (Invitrogen by Thermo Fisher Scientific). qPCR was performed using Fast Start TaqDNA Polymerase (Roche) and EvaGreen (Biotium), and gene expression was measured on the Lightcycler 480 (Roche). Expression was calculated as percentage relative to the average of housekeeping genes *GUSB*, *VPS29*, and *PSMB4*, which was set at 100%. All samples and genes were run in triplicate with 10 ng cDNA per reaction. The following primers were used: *PRAME* (forward: GTTGCTCAGGCACGTGGAT, reverse: CCCACTTAGAC TCAGGACACTTA), *HMHA1* (forward: GGTGCAGAGAATCCCG AGTT, reverse: CTGCTCCAGGAAGCTGAGG), *CD20* (forward: GGGGCTGTCCAGATTATGAA, reverse: GGAGTTTTTCTCCGTT GCTG), *WT1* (forward: AGACCCACACCAGGACTCAT, reverse: GATGCATGTTGTGATGGCGG), *GUSB* (forward: ACTGAACAGT CACCGACGAG, reverse: GGAACGCTGCACTTTTTGGT), *PSMB4* (forward: GTTTCCGCAACATCTCTCGC, reverse: CATCAATCA CCATCTGGCCG), *VPS29* (forward: TGAGAGGAGACTTCGATGA GAATC, reverse: TCTGCAACAGGGCTAAGCTG).

TCR gene transfer to healthy donor CD8+ T cells

The tumor-specific T cells were originally identified from leukemia patients after either HLA-matched allogeneic stem cell transplantation (allo-SCT) (HA-1H) or HLA-mismatched allo-SCT (PRAME),

or from the naive allo-HLA repertoire of healthy donors (CD20 and WT1).[17,19,20,24] Previously, the TCR sequences of the tumor-specific T cells and controls were identified by sequencing and the TCR chains were codon optimized, synthesized, and cloned in MP71-TCR-flex retroviral vectors by Baseclear. The MP71-TCR-flex vector contains codon-optimized and cysteine-modified murine TCRαβ constant domains and P2A sequence to link TCR chains, resulting in optimized TCR expression and increased preferential pairing.[45] Phoenix-AMPHO (ATCC) cells were transiently transfected with the created constructs and after 48 h retroviral supernatants were harvested and stored at −80°C. CD8+ T cells were isolated from PBMCs of an HLA-A*02:01-negative healthy individual, by MACS using anti-CD8 MicroBeads (Miltenyi Biotech/130-045-201). CD8+ T cells were stimulated with irradiated autologous feeders (40 Gy) and 0.8 μg/mL PHA in 24-well flat-bottom culture plates (Costar).

Two days after stimulation, we performed a knockout of the endogenous TCRα and TCRβ using CRISPR-Cas9, according to the previously described protocol.[23] In short, ribonuclear proteins (RNPs) were generated by complexing crRNA:trRNA (Integrated DNA Technologies [IDT]) with *Streptococcus pyogenes* Cas9 (IDT) as previously described.[46] TRAC-RNP (*TRAC* gRNA: TCAGGGTTCTGGATA TCTGT) and TRBC-RNP (*TRBC* gRNA: AGAGATCTCCCACAC CCAAA) were electroporated into CD8+ T cells using the NEON transfection system (Thermo Fisher Scientific) using transfection settings: 1600 V, 10 ms, three pulses.[47] Electroporated cells were immediately returned to fresh prewarmed TCM and incubated overnight. The next day, 3 days after stimulation, CD8+ T cells were transferred to 24-well flat-bottom suspension culture plates (Greiner Bio-One) for retroviral transduction. These plates were first coated with 30 μg/mL retronectin (Takara, Clontech) and blocked with 2% human serum albumin. Retroviral supernatants were added, and plates were centrifuged at 3000 × *g* for 20 min at 4°C. After removal of the retroviral supernatant, 0.3×10^6 CD8+ T cells were transferred per well. After O/N incubation, CD8+ T cells were transferred to 24-well flat-bottom culture plates (Costar). Seven days after stimulation, CD8+ T cells were MACS enriched for the murine TCR, using mTCR APC antibody (BD/553174) and anti-APC MicroBeads (Miltenyi Biotec/130-090-855). Ten days after stimulation, CD8+ T cells were functionally tested and purity was checked by flow cytometry.

Differentiation into hiPSC-derived cardiomyocytes and epicardial cells

Cardiomyocytes were induced as previously described, starting with $0.15–0.2 \times 10^6$ cells plated on Matrigel coated six-well culture plates in TeSR-E8 medium (Stem Cell Technologies) supplemented with 10 μM Rho-kinase inhibitor fasudil (LC Laboratories).[25] At day 0, the hiPSCs were cultured in BPEL medium[48] supplemented with a mixture of cytokines (20 ng/mL bone morphogenetic protein 4 [BMP4], R&D; 20 ng/mL ACTIVIN A, Miltenyi Biotec; 1.5 μM glycogen synthase kinase 3 inhibitor CHIR99021, Axon Medchem) to induce mesoderm formation in 3 days. Cytokines were removed and 5 μM WNT inhibitor (XAV939, TOCRIS) resulted in spontaneously contracting cardiomyocytes from day 6 onward. At day 12 of differentiation, media deprived of glucose and containing 4 mM sodium lactate (Sigma-Aldrich) finally stimulated cardiomyocyte enrichment in 4 days. Purity was determined by fluorescence-activated cell sorting (FACS) analysis of cardiac marker Troponin T. Mesoderm induction was also the starting point for the differentiation into epicardial cells.[26] Mesoderm was formed in 3 days, cytokines were removed, and a different mixture of BPEL medium with cytokines and small molecules was added (5 μM WNT inhibitor XAV939, TOCRIS; 1 μM retinoic acid [RA], Sigma-Aldrich; 30 ng/mL BMP4, R&D). At day 6, medium was refreshed with a mixture of 1 μM RA and 30 ng/mL BMP4 in BPEL medium. Finally, at day 9, cells were dissociated and seeded on 2.5 μg/mL fibronectin (bovine)-coated plates in BPEL medium supplemented with a transforming growth factor β (TGFβ) inhibitor (10 μM, SB431542, Tocris Bioscience). Epicardial cells were confluent after 4 days and differentiation purity was determined using a staining on epicardial marker T-box18.

Differentiation and hiPSC-derived kidney organoid formation

hiPSCs were plated on vitronectin-coated culture dishes at a concentration of 15,000 cells/cm^2 in Essential-8 medium (Life Technologies) supplemented with RevitaCell (Thermo Fisher Scientific). Differentiation was initiated the next day by culturing cells with 8 μM CHIR99021 (Tocris) in STEMdiff APEL-2 (Stem Cell Technologies) containing 1% PFHMII (Life Technologies) and 1% Antibiotic-Antimycotic (Life Technologies). On day 4, media was refreshed by APEL-2 medium supplemented with 200 ng/mL rhFGF9 (R&D Systems) and 1 mg/mL heparin (Sigma-Aldrich). On day 7, cells were pulsed for 1 h with 5 μM CHIR99021 in APEL-2, detached using Trypsin-EDTA (Thermo Fisher Scientific) and 3D culture formation was stimulated by transferring the cells as pellet containing 5×10^5 cells onto a transwell 0.4 mM pore polyester membrane (Corning). Cell pellets were cultured in the same medium. On day 7 + 5, growth factors were removed and organoids were cultured in APEL-2 medium without growth factors until day 7 + 18, changing medium every 2 days.[31]

T cell coculture

The T cell coculture approach varies for the three different target types, but all TCR-transduced T cells were produced similarly and added 10 days after stimulation. The tumor cell lines (30,000 cells) were directly cocultured with 5,000 T cells in 60 μL TCM in 384-well flat-bottom plates (Greiner Bio-One) and supernatant was harvested after 16 h. The hiPSC-derived heart cells were dissociated and transferred into 384-well flat-bottom plates (Greiner Bio-One) in two concentrations (5,000 and 10,000 cells per well). Both cell types were cultured in 60 μL TCM, only TGFβ inhibitor (10 μM) was added to the epicardial cells; 48 h later, enabling the hiPSCs to adhere to the bottom, 5,000 T cells were added per well and supernatant was harvested after 16 h. The hiPSC-derived kidney organoids were cocultured with 0.25×10^6 T cells per three organoids in one well. To stimulate infiltration, the T cells were resuspended in 50 μL APEL medium, pipetted directly on top of the organoids and incubated for 15 min. Afterward, 800 μL APEL medium was added on

top of the transwell and supernatant was harvested after 16 and 40 h of incubation.

IFN-γ production

After coculture, the supernatants were harvested in 384-well flat-bottom plates (Greiner Bio-One) and IFN-γ production levels were measured by an IFN-γ ELISA (Sanquin). During the ELISA procedure, supernatants were transferred using the Hamilton Microlab STAR Liquid Handling System (Hamilton company) and diluted 1:5, 1:25, and/or 1:125 to quantify IFN-γ production levels within the area of the standard curve.

Dissociation of the hiPSC-derived kidney organoids

After 40 h of coculture, the kidney organoids were dissociated using an enzymatic and mechanical approach.[49] Single organoids were incubated for 40 min at 37°C in a collagenase buffer containing 600 U mL^{-1} collagenase Type I (Worthington) and 0.75 U mL^{-1} DNAse (Sigma-Aldrich) in $HBSS^{+/+}$ (with calcium and magnesium; Thermo Fisher Scientific). Every 10 min the dissociation was further stimulated by resuspending the cells. Cells were centrifuged and incubated for 5 min at 37°C in a TrypLE buffer containing 5 U mL^{-1} DNAse I (Sigma-Aldrich) and 4 μg mL^{-1} heparin (Sigma-Aldrich) in 80% TrypLE Select 10x (Thermo Fisher Scientific) in DPBS (Thermo Fisher Scientific). The dissociation was further stimulated by resuspending the cells and thereafter directly stopped by adding $HBSS^{+/+}$ with 10% FCS. Cells were centrifuged and collected for flow cytometry in 1 mL PBS +0.1% BSA.

Flow cytometry

FACS was performed on an LSR II flow cytometer (BD Biosciences) and data were analyzed using FlowJo software (TreeStar). T cells, the non-adherent hiPSC-derived heart cells and dissociated hiPSC-derived kidney organoid cells were washed and stained for 30 min at 4°C (Table S1). pMHC-multimers were synthesized as previously described.[19] For the pMHC-multimer staining, CD8 cells were 1:1 mixed with the K562 cell line to prevent the formation of large aggregates. Cells were stained for 10 min at 37°C with the PE-conjugated pMHC-multimers, directly followed by an AF700-conjugated CD8 antibody for 15 min at 4°C. For the CD107a staining, antibodies were added at the start of the coculture, in order to detect the total transient expression on the surface during the coculture period.

Immunofluorescence hiPSC-derived heart cells

After coculture and collection of the non-adherent hiPSC-derived heart cells, the remaining adherent cells in the 384-well test plates were fixed with 2% PFA for 30 min at room temperature (RT) and stained with 1 μM DAPI for 5 min at RT. Cells were washed with PBS following both steps. Fluorescent images were captured with the EVOS FL AUTO2 microscope, using a ×10 magnification objective. Six images were taken per well, covering in total 50% of the wells. The number of DAPI-positive cells per image was calculated via cell segmentations with the free open-source software CellProfiler.[50] The percentage of killed cells was quantified by comparing the number of cells in the test wells with the average number of cells in the control wells without T cells. For each image, the following formula was used: % killing = (1 − (number of cells in the test well/average number of cells in the control well)) *100.

Immunofluorescence hiPSC-derived kidney organoids

hiPSC-derived kidney organoids were fixed in 2% paraformaldehyde (PFA) at 4°C for 20 min and washed three times in PBS. Prior to staining, organoids were permeabilized and blocked for 2 h at RT in PBS with 0.3% Triton X and 10% donkey serum. Primary antibodies were incubated overnight at 4°C and secondary antibodies for 2 h at RT (Table S2). Organoids were counterstained with Hoechst33258 for 5 min at RT. The antibodies were diluted in PBS with 0.3% Triton X- and 10% donkey serum and organoids were washed three times in PBS after each step. Finally, organoids were embedded in ProLong Gold Antifade Mountant (Thermo Fisher Scientific) in 35-mm glass bottom dishes (MatTek corporation). Images were taken with the Andor Dragonfly 200 Confocal Microscope, using Fusion software. Final images were processed with the ImageJ software.

Ethics approval

This study involves materials from human participants and was approved by Institutional Review Board of the Leiden University Medical Center (approval number 3.4205/010/FB/jr) and the METC-LDD (approval number HEM 008/SH/sh). Materials were collected after written informed consent.

DATA AVAILABILITY

All data relevant to the study are included in the article or uploaded as supplementary information.

SUPPLEMENTAL INFORMATION

Supplemental information can be found online at https://doi.org/10.1016/j.omtm.2023.01.005.

ACKNOWLEDGMENTS

The authors thank the CCB Light Microscopy Facility for equipment and assistance (LUMC, The Netherlands), Ellen Lievers (Department of Nephrology, LUMC, the Netherlands) for technical assistance at stem cell and organoid culture and immunofluorescence staining, Marije Koning (Department of Nephrology, LUMC, the Netherlands) for the kidney organoid dissociation protocol, and Amy Cochrane (Department of Anatomy and Embryology, LUMC, the Netherlands) for capturing fluorescent DAPI images and performing cell segmentations. The research in this study was funded by Health-Holland (grant number LSHM15011) and Bellicum Pharmaceuticals (unrestricted grant). The graphical abstract was created using https://biorender.com.

AUTHOR CONTRIBUTIONS

R.A.v.A. designed, performed, analyzed, and interpreted all experiments and wrote the manuscript. L.T.M. performed the TCRαβ knockouts. U.G.C. generated the hiPSC-derived heart cells. D.F.G.R. performed qPCR. R.S.H. constructed retroviral expression

vectors. C.W.v.d.B. generated the hiPSC-derived kidney organoids and designed and interpreted experiments. C.F. generated the hiPSC-derived heart cells and designed and interpreted experiments. J.H.F.F. supervised the study and revised the manuscript. M.H.M.H. designed and supervised the study, interpreted the experiments and wrote the manuscript. All authors reviewed the manuscript.

DECLARATION OF INTERESTS

Miltenyi Biotec has licensed the PRAME and HA1 TCR.

REFERENCES

1. Zhang, J., and Wang, L. (2019). The emerging world of TCR-T cell trials against cancer: a systematic Review. Technol. Cancer Res. Treat. *18*.
2. Linnemann, C., Schumacher, T.N.M., and Bendle, G.M. (2011). T-cell receptor gene therapy: critical parameters for clinical success. J. Invest. Dermatol. *131*, 1806–1816.
3. Rath, J.A., and Arber, C. (2020). Engineering strategies to enhance TCR-based adoptive T cell therapy. Cells *9*, 1485.
4. D'Ippolito, E., Schober, K., Nauerth, M., and Busch, D.H. (2019). T cell engineering for adoptive T cell therapy: safety and receptor avidity. Cancer Immunol. Immunother. *68*, 1701–1712.
5. Karpanen, T., and Olweus, J. (2015). T-cell receptor gene therapy – ready to go viral? Mol. Oncol. *9*, 2019–2042.
6. Morgan, R.A., Chinnasamy, N., Abate-Daga, D., Gros, A., Robbins, P.F., Zheng, Z., Dudley, M.E., Feldman, S.A., Yang, J.C., Sherry, R.M., et al. (2013). Cancer regression and neurological toxicity following anti-MAGE-A3 TCR gene therapy. J. Immunother. *36*, 133–151.
7. Johnson, L.A., Heemskerk, B., Powell, D.J., Jr., Cohen, C.J., Morgan, R.A., Dudley, M.E., Robbins, P.F., and Rosenberg, S.A. (2006). Gene transfer of tumor-reactive TCR confers both high avidity and tumor reactivity to nonreactive peripheral blood mononuclear cells and tumor-infiltrating lymphocytes. J. Immunol. *177*, 6548–6559.
8. Parkhurst, M.R., Yang, J.C., Langan, R.C., Dudley, M.E., Nathan, D.A.N., Feldman, S.A., Davis, J.L., Morgan, R.A., Merino, M.J., Sherry, R.M., et al. (2011). T cells targeting carcinoembryonic antigen can mediate regression of metastatic colorectal cancer but induce severe transient colitis. Mol. Ther. *19*, 620–626.
9. Takasato, M., Er, P.X., Chiu, H.S., Maier, B., Baillie, G.J., Ferguson, C., Parton, R.G., Wolvetang, E.J., Roost, M.S., Chuva de Sousa Lopes, S.M., and Little, M.H. (2015). Kidney organoids from human iPS cells contain multiple lineages and model human nephrogenesis. Nature *526*, 564–568.
10. Talman, V., and Kivelä, R. (2018). Cardiomyocyte-endothelial cell interactions in cardiac remodeling and regeneration. Front. Cardiovasc. Med. *5*, 101.
11. Rowe, R.G., and Daley, G.Q. (2019). Induced pluripotent stem cells in disease modelling and drug discovery. Nat. Rev. Genet. *20*, 377–388.
12. Freund, C., and Mummery, C.L. (2009). Prospects for pluripotent stem cell-derived cardiomyocytes in cardiac cell therapy and as disease models. J. Cell. Biochem. *107*, 592–599.
13. Yuki, K., Cheng, N., Nakano, M., and Kuo, C.J. (2020). Organoid models of tumor immunology. Trends Immunol. *41*, 652–664.
14. Dijkstra, K.K., Cattaneo, C.M., Weeber, F., Chalabi, M., van de Haar, J., Fanchi, L.F., Slagter, M., van der Velden, D.L., Kaing, S., Kelderman, S., et al. (2018). Generation of tumor-reactive T cells by Co-culture of peripheral blood lymphocytes and tumor organoids. Cell *174*, 1586–1598.e12.
15. Schnalzger, T.E., de Groot, M.H., Zhang, C., Mosa, M.H., Michels, B.E., Röder, J., Darvishi, T., Wels, W.S., and Farin, H.F. (2019). 3D model for CAR-mediated cytotoxicity using patient-derived colorectal cancer organoids. EMBO J. *38*, e100928.
16. van Balen, P., Jedema, I., van Loenen, M.M., de Boer, R., van Egmond, H.M., Hagedoorn, R.S., Hoogstaten, C., Veld, S.A.J., Hageman, L., van Liempt, P.A.G., et al. (2020). HA-1H T-cell receptor gene transfer to redirect virus-specific T cells for treatment of hematological malignancies after allogeneic stem cell transplantation: a phase 1 clinical study. Front. Immunol. *11*, 1804.
17. Amir, A.L., van der Steen, D.M., van Loenen, M.M., Hagedoorn, R.S., de Boer, R., Kester, M.D.G., de Ru, A.H., Lugthart, G.J., van Kooten, C., Hiemstra, P.S., et al. (2011). PRAME-specific Allo-HLA-restricted T cells with potent antitumor reactivity useful for therapeutic T-cell receptor gene transfer. Clin. Cancer Res. *17*, 5615–5625.
18. Orlando, D., Miele, E., De Angelis, B., Guercio, M., Boffa, I., Sinibaldi, M., Po, A., Caruana, I., Abballe, L., Carai, A., et al. (2018). Adoptive immunotherapy using PRAME-specific T cells in medulloblastoma. Cancer Res. *78*, 3337–3349.
19. Jahn, L., van der Steen, D.M., Hagedoorn, R.S., Hombrink, P., Kester, M.G.D., Schoonakker, M.P., de Ridder, D., van Veelen, P.A., Falkenburg, J.H.F., and Heemskerk, M.H.M. (2016). Generation of CD20-specific TCRs for TCR gene therapy of CD20low B-cell malignancies insusceptible to CD20-targeting antibodies. Oncotarget *7*, 77021–77037.
20. van Amerongen, R.A., Hagedoorn, R.S., Remst, D.F.G., Assendelft, D.C., van der Steen, D.M., Wouters, A.K., van de Meent, M., Kester, M.G.D., de Ru, A.H., Griffioen, M., et al. (2022). WT1-specific TCRs directed against newly identified peptides install antitumor reactivity against acute myeloid leukemia and ovarian carcinoma. J. Immunother. Cancer *10*, e004409.
21. Amir, A.L., van der Steen, D.M., Hagedoorn, R.S., Kester, M.G.D., van Bergen, C.A.M., Drijfhout, J.W., de Ru, A.H., Falkenburg, J.H.F., van Veelen, P.A., and Heemskerk, M.H.M. (2011). Allo-HLA-reactive T cells inducing graft-versus-host disease are single peptide specific. Blood *118*, 6733–6742.
22. Heemskerk, M.H.M., Hagedoorn, R.S., van der Hoorn, M.A.W.G., van der Veken, L.T., Hoogeboom, M., Kester, M.G.D., Willemze, R., and Falkenburg, J.H.F. (2007). Efficiency of T-cell receptor expression in dual-specific T cells is controlled by the intrinsic qualities of the TCR chains within the TCR-CD3 complex. Blood *109*, 235–243.
23. Morton, L.T., Reijmers, R.M., Wouters, A.K., Kweekel, C., Remst, D.F.G., Pothast, C.R., Falkenburg, J.H.F., and Heemskerk, M.H.M. (2020). Simultaneous deletion of endogenous TCRalphabeta for TCR gene therapy creates an improved and safe cellular therapeutic. Mol. Ther. *28*, 64–74.
24. Marijt, W.A.E., Heemskerk, M.H.M., Kloosterboer, F.M., Goulmy, E., Kester, M.G.D., van der Hoorn, M.A.W.G., van Luxemburg-Heys, S.A.P., Hoogeboom, M., Mutis, T., Drijfhout, J.W., et al. (2003). Hematopoiesis-restricted minor histocompatibility antigens HA-1- or HA-2-specific T cells can induce complete remissions of relapsed leukemia. Proc. Natl. Acad. Sci. USA *100*, 2742–2747.
25. Zhang, M., D'Aniello, C., Verkerk, A.O., Wrobel, E., Frank, S., Ward-van Oostwaard, D., Piccini, I., Freund, C., Rao, J., Seebohm, G., et al. (2014). Recessive cardiac phenotypes in induced pluripotent stem cell models of Jervell and Lange-Nielsen syndrome: disease mechanisms and pharmacological rescue. Proc. Natl. Acad. Sci. USA *111*, E5383–E5392.
26. Guadix, J.A., Orlova, V.V., Giacomelli, E., Bellin, M., Ribeiro, M.C., Mummery, C.L., Pérez-Pomares, J.M., and Passier, R. (2017). Human pluripotent stem cell differentiation into functional epicardial progenitor cells. Stem Cell Rep. *9*, 1754–1764.
27. Wilm, B., and Muñoz-Chapuli, R. (2016). The role of WT1 in embryonic development and normal organ homeostasis. Methods Mol. Biol. *1467*, 23–39.
28. Hastie, N.D. (2017). Wilms' tumour 1 (WT1) in development, homeostasis and disease. Development *144*, 2862–2872.
29. Duim, S.N., Goumans, M.J., and Kruithof, B.P.T. (2016). WT1 in cardiac development and disease. In *Wilms Tumor*. Brisbane (AU), M.M. van den Heuvel-Eibrink, ed. (Codon Publications Copyright: The Authors.).
30. Madeira, F., Park, Y.M., Lee, J., Buso, N., Gur, T., Madhusoodanan, N., Basutkar, P., Tivey, A.R.N., Potter, S.C., Finn, R.D., and Lopez, R. (2019). The EMBL-EBI search and sequence analysis tools APIs in 2019. Nucleic Acids Res. *47*, w636–w641.
31. van den Berg, C.W., Ritsma, L., Avramut, M.C., Wiersma, L.E., van den Berg, B.M., Leuning, D.G., Lievers, E., Koning, M., Vanslambrouck, J.M., Koster, A.J., et al. (2018). Renal subcapsular transplantation of PSC-derived kidney organoids induces neo-vasculogenesis and significant glomerular and tubular maturation in vivo. Stem Cell Rep. *10*, 751–765.
32. Betts, M.R., Brenchley, J.M., Price, D.A., De Rosa, S.C., Douek, D.C., Roederer, M., and Koup, R.A. (2003). Sensitive and viable identification of antigen-specific CD8+ T cells by a flow cytometric assay for degranulation. J. Immunol. Methods *281*, 65–78.
33. Wolfl, M., Kuball, J., Ho, W.Y., Nguyen, H., Manley, T.J., Bleakley, M., and Greenberg, P.D. (2007). Activation-induced expression of CD137 permits detection,

isolation, and expansion of the full repertoire of CD8+ T cells responding to antigen without requiring knowledge of epitope specificities. Blood *110*, 201–210.

34. Lebedeva, T., Dustin, M.L., and Sykulev, Y. (2005). ICAM-1 co-stimulates target cells to facilitate antigen presentation. Curr. Opin. Immunol. *17*, 251–258.

35. Riesbeck, K., Billström, A., Tordsson, J., Brodin, T., Kristensson, K., and Dohlsten, M. (1998). Endothelial cells expressing an inflammatory phenotype are lysed by superantigen-targeted cytotoxic T cells. Clin. Diagn. Lab. Immunol. *5*, 675–682.

36. Mickelson, J.K., Kukielka, G., Bravenec, J.S., Mainolfi, E., Rothlein, R., Hawkins, H.K., Kelly, J.H., and Smith, C.W. (1995). Differential expression and release of CD54 induced by cytokines. Hepatology *22*, 866–875.

37. Janeway, C.A., Travers, P., Walport, M., and Shlomchik, M.J. (2001). Immunobiology: The Immune System in Health and Disease, 5th edition (Garland Science).

38. Cameron, B.J., Gerry, A.B., Dukes, J., Harper, J.V., Kannan, V., Bianchi, F.C., Grand, F., Brewer, J.E., Gupta, M., Plesa, G., et al. (2013). Identification of a titin-derived HLA-A1–presented peptide as a cross-reactive target for engineered MAGE A3–directed T cells. Sci. Transl. Med. *5*, 197ra103.

39. van den Berg, C.W., Elliott, D.A., Braam, S.R., Mummery, C.L., and Davis, R.P. (2016). Differentiation of human pluripotent stem cells to cardiomyocytes under defined conditions. Methods Mol. Biol. *1353*, 163–180.

40. Campostrini, G., Meraviglia, V., Giacomelli, E., van Helden, R.W.J., Yiangou, L., Davis, R.P., Bellin, M., Orlova, V.V., and Mummery, C.L. (2021). Generation, functional analysis and applications of isogenic three-dimensional self-aggregating cardiac microtissues from human pluripotent stem cells. Nat. Protoc. *16*, 2213–2256.

41. Tawara, I., Kageyama, S., Miyahara, Y., Fujiwara, H., Nishida, T., Akatsuka, Y., Ikeda, H., Tanimoto, K., Terakura, S., Murata, M., et al. (2017). Safety and persistence of WT1-specific T-cell receptor gene-transduced lymphocytes in patients with AML and MDS. Blood *130*, 1985–1994.

42. Chapuis, A.G., Egan, D.N., Bar, M., Schmitt, T.M., McAfee, M.S., Paulson, K.G., Voillet, V., Gottardo, R., Ragnarsson, G.B., Bleakley, M., et al. (2019). T cell receptor gene therapy targeting WT1 prevents acute myeloid leukemia relapse post-transplant. Nat. Med. *25*, 1064–1072.

43. Di Stasi, A., Jimenez, A.M., Minagawa, K., Al-Obaidi, M., and Rezvani, K. (2015). Review of the results of WT1 peptide vaccination strategies for myelodysplastic syndromes and acute myeloid leukemia from nine different studies. Front. Immunol. *6*, 36.

44. van der Wal, E., Bergsma, A.J., van Gestel, T.J.M., Zaehres, H., Araúzo-Bravo, M.J., Schöler, H.R., and van der Ploeg, A.T. (2017). Pijnappel WWMP. GAA deficiency in pompe disease is alleviated by exon inclusion in iPSC-derived skeletal muscle cells. Mol. Ther. Nucleic Acids *7*, 101–115.

45. Linnemann, C., Heemskerk, B., Kvistborg, P., Kluin, R.J.C., Bolotin, D.A., Chen, X., Bresser, K., Nieuwland, M., Schotte, R., Michels, S., et al. (2013). High-throughput identification of antigen-specific TCRs by TCR gene capture. Nat. Med. *19*, 1534–1541.

46. Seki, A., and Rutz, S. (2018). Optimized RNP transfection for highly efficient CRISPR/Cas9-mediated gene knockout in primary T cells. J. Exp. Med. *215*, 985–997.

47. Schumann, K., Lin, S., Boyer, E., Simeonov, D.R., Subramaniam, M., Gate, R.E., Haliburton, G.E., Ye, C.J., Bluestone, J.A., Doudna, J.A., and Marson, A. (2015). Generation of knock-in primary human T cells using Cas9 ribonucleoproteins. Proc. Natl. Acad. Sci. USA *112*, 10437–10442.

48. Ng, E.S., Davis, R., Stanley, E.G., and Elefanty, A.G. (2008). A protocol describing the use of a recombinant protein-based, animal product-free medium (APEL) for human embryonic stem cell differentiation as spin embryoid bodies. Nat. Protoc. *3*, 768–776.

49. Koning, M., Dumas, S.J., Avramut, M.C., Koning, R.I., Meta, E., Lievers, E., Wiersma, L.E., Borri, M., Liang, X., Xie, L., et al. (2022). Vasculogenesis in kidney organoids upon transplantation. NPJ Regen. Med. *7*, 40.

50. Carpenter, A.E., Jones, T.R., Lamprecht, M.R., Clarke, C., Kang, I.H., Friman, O., Guertin, D.A., Chang, J.H., Lindquist, R.A., Moffat, J., et al. (2006). CellProfiler: image analysis software for identifying and quantifying cell phenotypes. Genome Biol. *7*, R100.

OMTM, Volume *28*

Supplemental information

Human iPSC-derived preclinical models to identify toxicity of tumor-specific T cells with clinical potential

Rosa A. van Amerongen, Laura T. Morton, Umesh G. Chaudhari, Dennis F.G. Remst, Renate S. Hagedoorn, Cathelijne W. van den Berg, Christian Freund, J.H. Frederik Falkenburg, and Mirjam H.M. Heemskerk

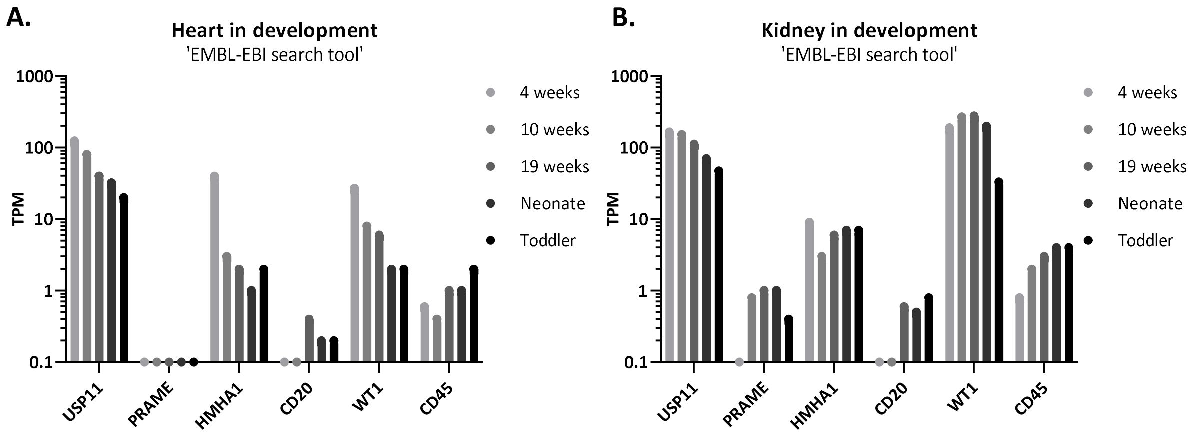

Figure S1: Expression of the T-cell targets in kidney and heart during development

Public available RNAseq gene expression data of the T-cell targets in heart **(A)** and kidney **(B)** tissue during development, given in transcripts per million (TPM). The data is extracted from the online available EMBL-EBI search tool in dataset: 'Human RNA-seq time-series of the development of seven major organs'.[30] Two or three samples are shown per time frame, with three timepoints after fertilization (4, 10 and 19 weeks), one neonatal timepoint (heart samples = 0, 6 or 94 days, kidney samples = 0, 18 or 34 days) and one toddler timepoint (heart and kidney samples = 2 and 4 years). Since HMHA1 and CD20 are hematopoietic-restricted marker, expression of the hematopoietic-restricted marker CD45 is included as a measure for the presence of hematopoietic cells in these samples.

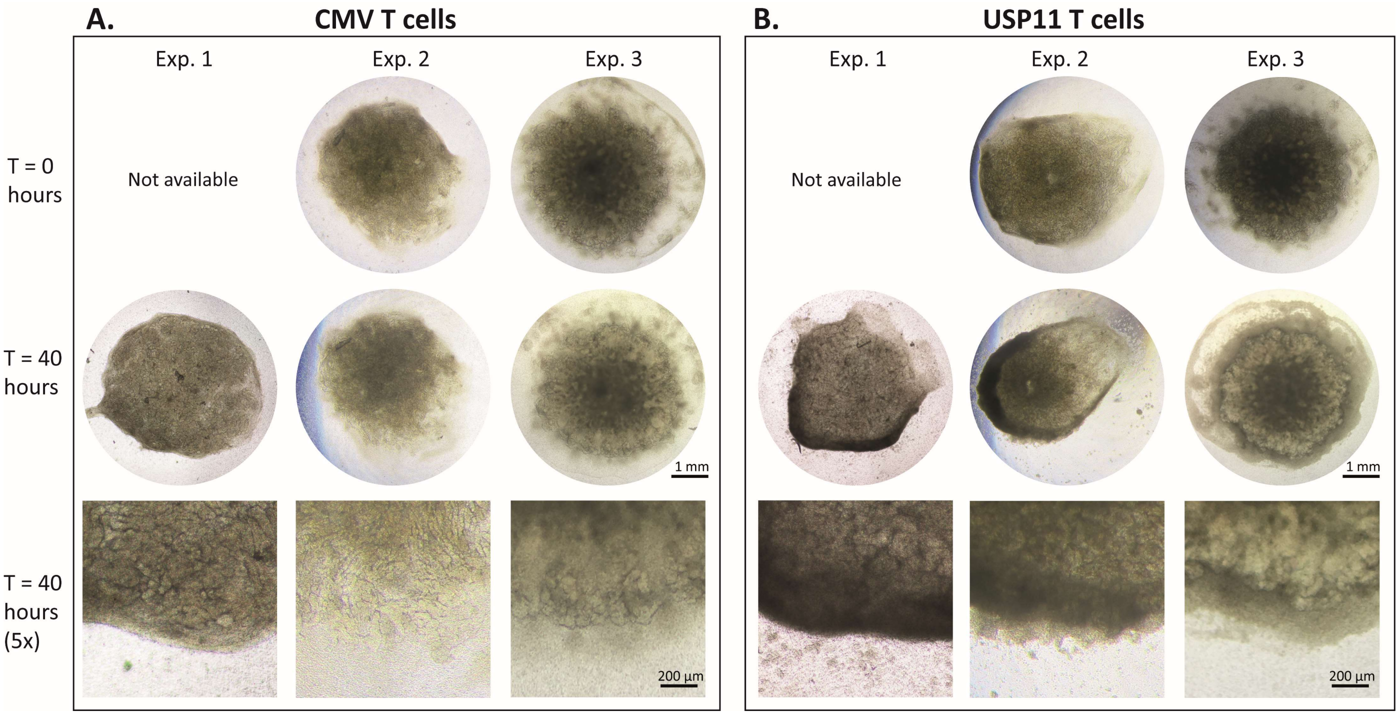

Figure S2: Transmission light images of organoids cocultured with CMV and USP11 T cells

Transmission light images of organoids at timepoint 0 and after 40 hours of coculture with CMV T cells (**A**) or USP11 T cells (**B**). Shown are representative images for three independent experiments (Exp.). Per experiment three organoids were included for each T-cell product. The 5x images shown for T = 40 zoom in on the edges of the organoids.

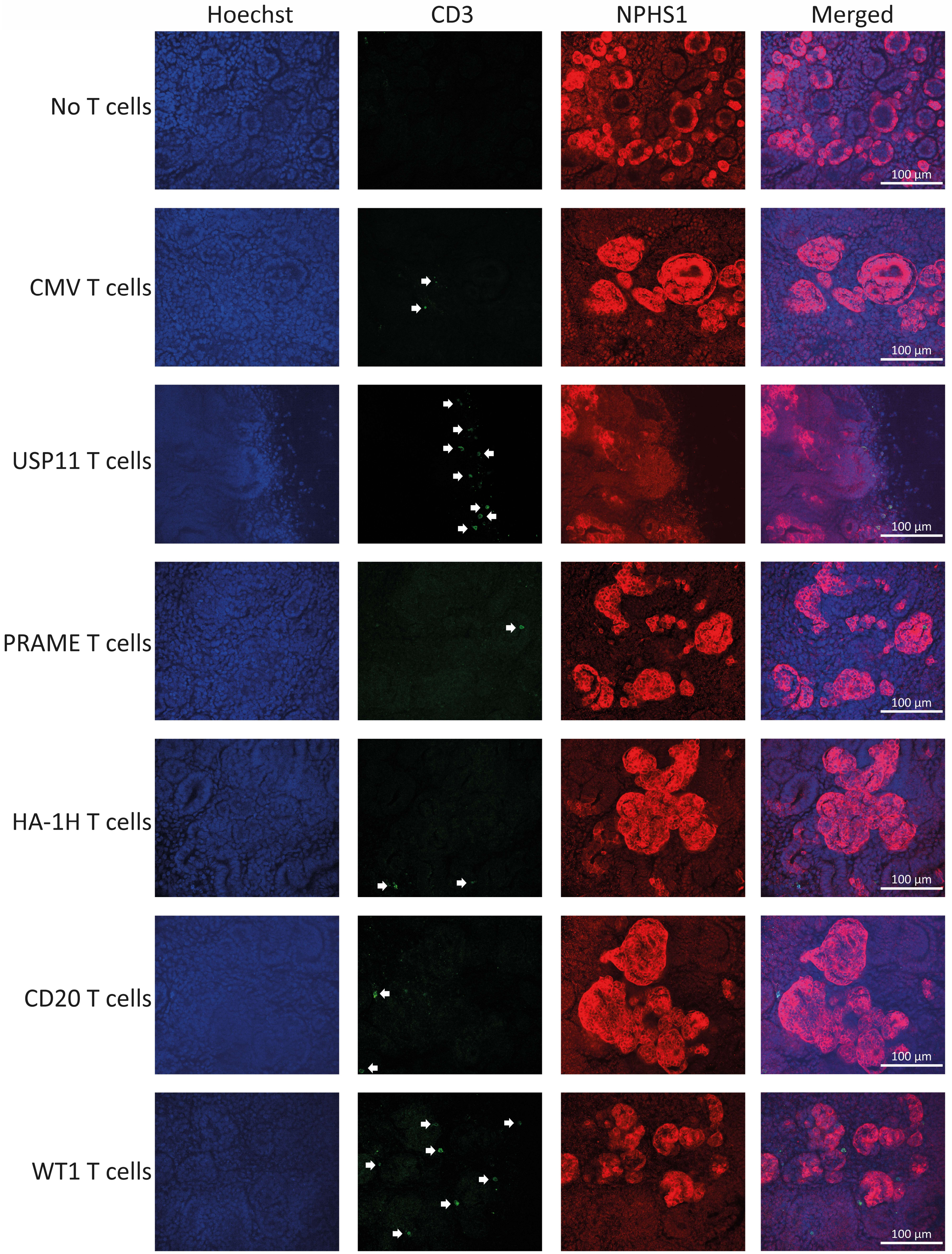

Figure S3: Immunofluorescent images of the kidney organoids cultured with the different T-cell products

Immunofluorescent images of the organoids after 40 hours of coculture with the different T cells, showing glomerular structures (NPHS1), T cells (CD3) and a counterstaining with Hoechst (nuclei). White arrows mark T cells. The images show a variation in number of infiltrated T cells and location of the T cells. 10 images were captured per organoid, representative images are shown.

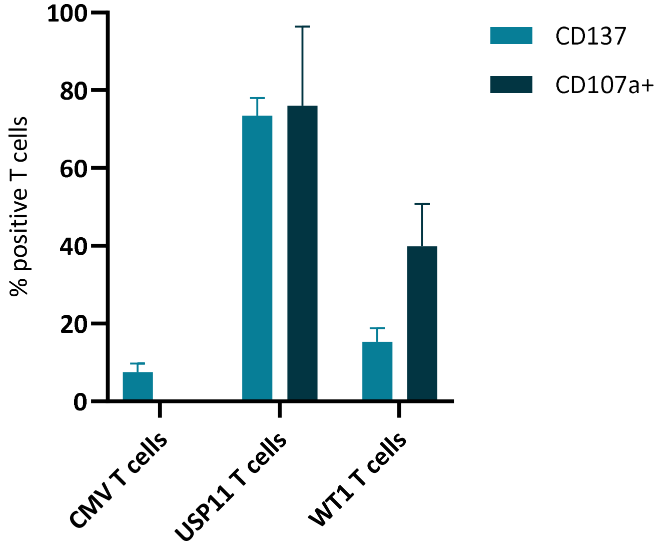

Figure S4: CD137 and CD107a expression on CD8+ T cells infiltrated in hiPSC-derived kidney organoids

The combined percentages of CD137 and CD107a positive infiltrated CD8+ T cells after 40 hours of coculture with the hiPSC-derived kidney organoids. Shown is data of one experiment, two organoids were dissociated for each T-cell product. Values and error bars represent mean and SD of the two organoids.

Table S1. Antibodies used for flow cytometry

Antibody	Conjugate	Company	Article number	Dilution
CD8	Alexa Fluor 700	Invitrogen	MHCD0829	1:300
Murine TCR-Cβ	APC	BD Pharmingen	553174	1:300
Human TCR-αβ	PerCP-Cy5.5	BioLegend	306724	1:150
pMHC-multimers	PE	In house	x	1:300
CD8	PE	BD Pharmingen	555367	1:960
CD137	APC	BD Pharmingen	550890	1:60
CD107a	BV 421	BD Horizon	562623	1:28
CD54	APC	BioLegend	353112	1:240
HLA-A*02:01	PE	BD Pharmingen	558570	1:300
Cardiac Troponin T	VioBlue	Miltenyi Biotec	130-120-402	1:50
T-box18 (IgG)	x	Sigma-Aldrich	HPA029014	1:100
IgG antibody	Alexa Fluor 488	Invitrogen	A11034	1:200

Table S2. Antibodies used for immunofluorescence of hiPSC-derived kidney organoids

Antibody name	Conjugate/Species	Company	Article number	Dilution
NPHS1	Sheep	R&D Systems	AF4269	1:100
CD3	Mouse	Dako	M7254	1:25
Hoechst33258	x	Thermo Fisher Scientific	H3570	1:10,000
Donkey-α-Sheep	Alexa Fluor 647	Thermo Fisher Scientific	A-21448	1:500
Donkey-α-Mouse	Alexa Fluor 488	Thermo Fisher Scientific	A-21202	1:500
